# Supplementary material for: Spongy all-in-liquid materials by in-situ formation of emulsions at oil-water interfaces
Source: Nat Commun. 2022 Jul 18;13:4162. doi: 10.1038/s41467-022-31644-2 (PMC9293904; doi:10.1038/s41467-022-31644-2)
Supplement: Supplementary file 1 — Supplementary Information [file 41467_2022_31644_MOESM1_ESM.pdf]

Supplementary Information: Spongy all-in-liquid materials by in-situ formation of emulsions at oil-water interfaces

Parisa Bazazi[1,*], Howard A. Stone[2], and S. Hossein Hejazi[1,*]

[1] *Department of Chemical and Petroleum Engineering, University of Calgary, Calgary, AB T2N 1N, Canada*

[2] *Department of Mechanical and Aerospace Engineering Princeton University, Princeton, NJ 08544, USA*

[*] *Corresponding authors: parisa.bazazi@ucalgary.ca, shhejazi@ucalgary.ca*

Supplementary Note 1: Interfacial tension and viscosity of micellar solutions

The interfacial tension of silica dispersion-Span micellar solutions is measured using a spinning drop method as explained in the Methods Section and data are presented in **Fig. S1a** and **Fig. S2**. The shear viscosity of Span micellar solutions, measured using a Discovery Hybrid Rheometer, is reported in **Fig. S1b**, where all solutions exhibit shear thinning behavior. In these systems, the viscosity is evaluated at the average shear rate at each injection speed. The range of shear rates in the experiments, calculated as $8U/d_i$, where U and d_i are the injection speed and the inner diameter of the needle [1], is highlighted in green in **Fig. S1b**.

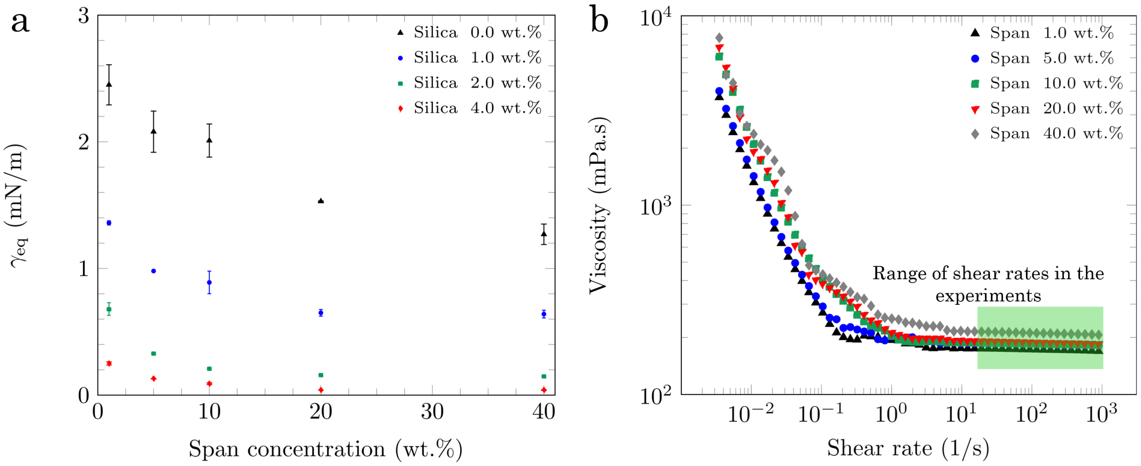

Figure S1: **(a)** Interfacial tension of silica dispersion-Span micellar solutions and **(b)** viscosity of micellar solutions. The error bars represent the standard deviation of three measurements. Source data are provided as a Source Data file.

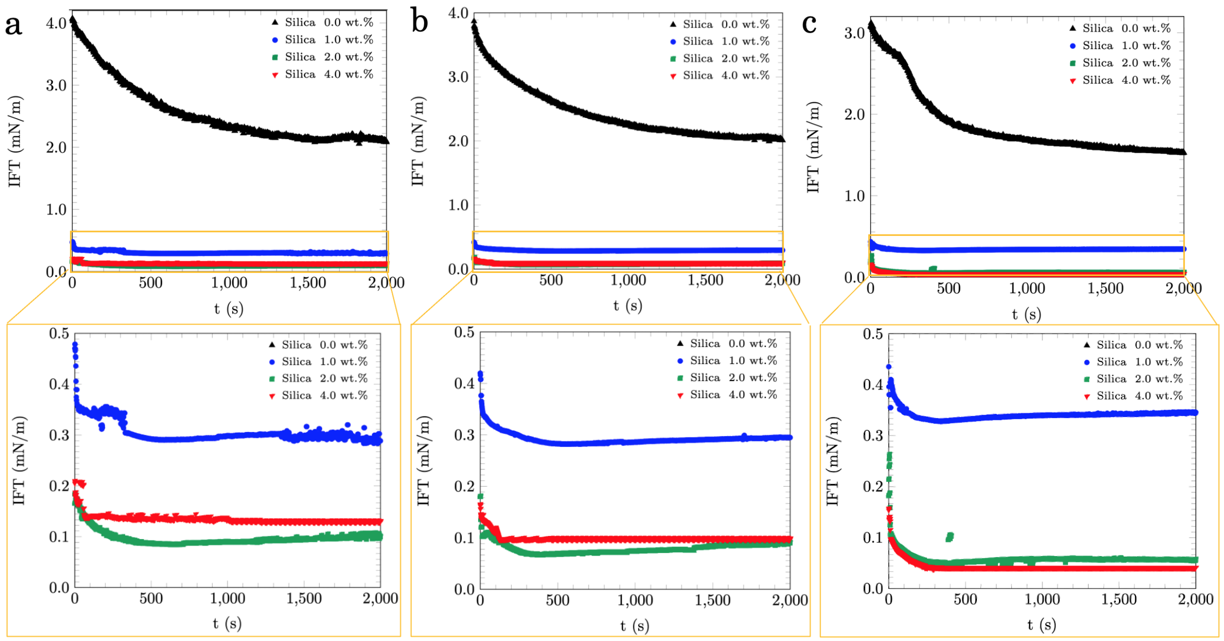

Figure S2: Dynamic interfacial tension. **(a)** Span 5.0 wt.%, **(b)** Span 10.0 wt.%, and **(c)** Span 20.0 wt.%. Source data are provided as a Source Data file.

Supplementary Note 2: Morphology of liquid jets

The aqueous phase (DI water or silica dispersions at concentrations of 1.0, 2.0, and 4.0 wt.%) is injected (average speeds of $8.0 \times 10^{-4} - 1.1 \times 10^{-1}$ m/s) into a reservoir of Span 80 micellar solutions at concentrations of 1.0, 5.0, 10.0, 20.0 and 40.0 wt.%. The oil viscosity is fixed at 135 mPa.s, while the addition of Span increases the oil viscosity up to 257 mPa.s at 40.0 wt.% Span (**Fig. S1**). We observe four flow regimes, droplet, bead-on-a-string (BOAS), column, and connected, which are identified by border lines colored, respectively, in black, blue, green, and red in (**Figs. S3-S6**).

For the case of DI water, single droplets detach from the needle when Q < 10 μl/s whereas they detach at a distance of $1 - 2$ mm away from the needle tip for Q ≥ 10 μl/s, and sediment

through the micellar solution reservoir (**Fig. S3**). The injection of 1.0 wt.% silica dispersion into micellar solutions generates three flow regimes. In 1.0 and 5.0 wt.% Span micellar solutions, only the droplet state is observed, while in 10.0, 20.0, and 40.0 wt.% Span micellar solutions, BOAS ($Q < 10\ \mu l/s$) and connected ($Q \geq 10\ \mu l/s$) regimes are identified (**Fig. S4**). The flow regimes from the injection of 2.0 wt.% silica dispersion into 1.0, 5.0, 10.0, and 20.0 wt.% Span micellar solutions are similar to those of a 1.0 wt.% silica dispersion. However, flow morphologies significantly change at 40.0 wt.% Span micellar solution. At this Span concentration, in addition to the BOAS state (low injection flow rates) and connected droplet state (high injection flow rates), straight liquid pipes, i.e. liquid columns, are formed at intermediate flow rates (0.3 - 1.0 μl/s, **Fig. S5**). Increasing the silica concentration to 4.0 wt.%, results in the formation of stable liquid columns in a wider range of Span concentrations (10-40 wt.%) and injection flow rates ($0.1 - 4.0\ \mu$l/s, **Fig. S6**).

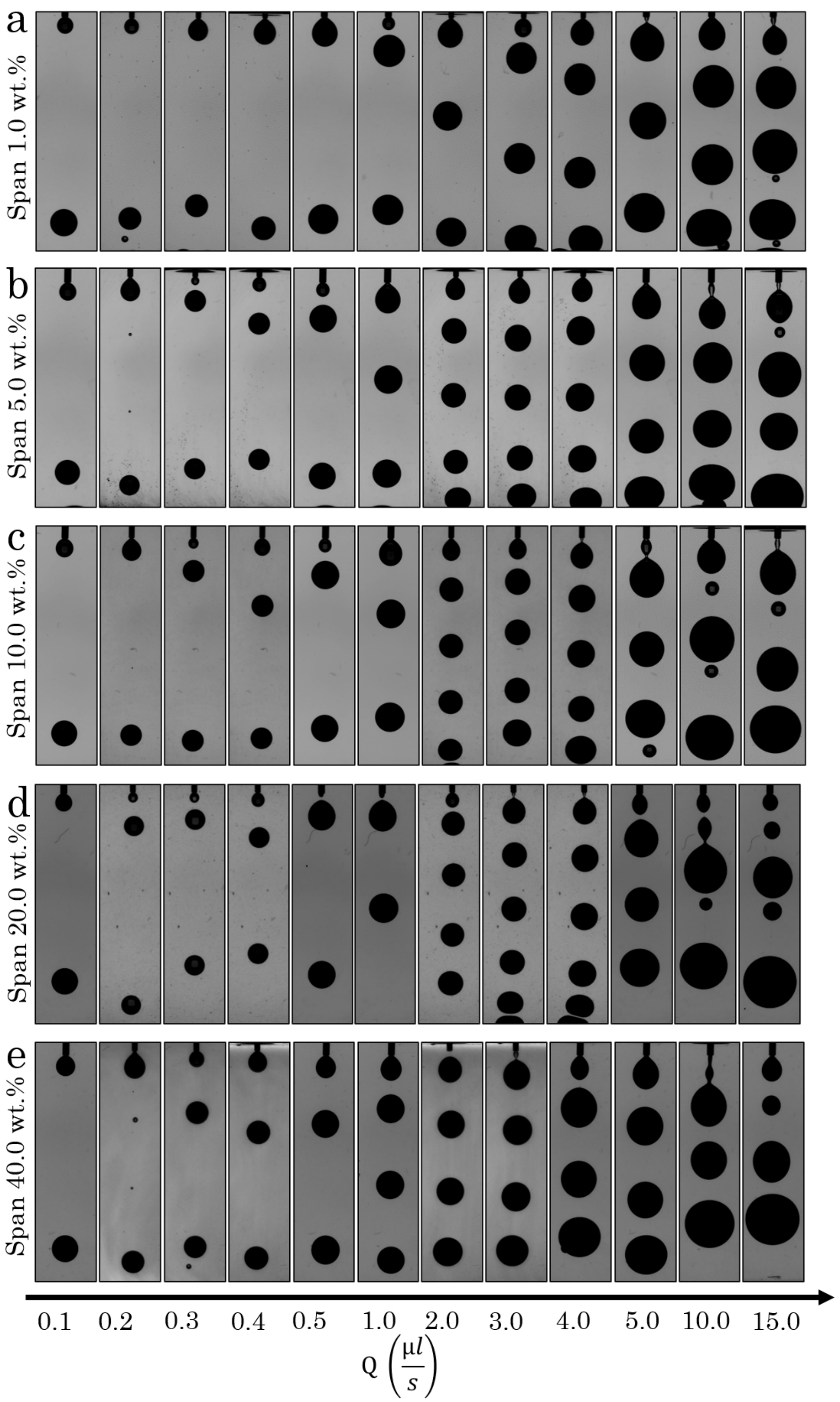

Figure S3: Injection of DI water into Span micellar solutions (viscosity of mineral oil = 135 $mPa.s$) at concentrations of **(a)** 1.0 wt.%, **(b)** 5.0 wt.%, **(c)** 10.0 wt.%, **(d)** 20.0 wt.%, and **(e)** 40.0 wt.%. The diameter of the needle is 500 μm.

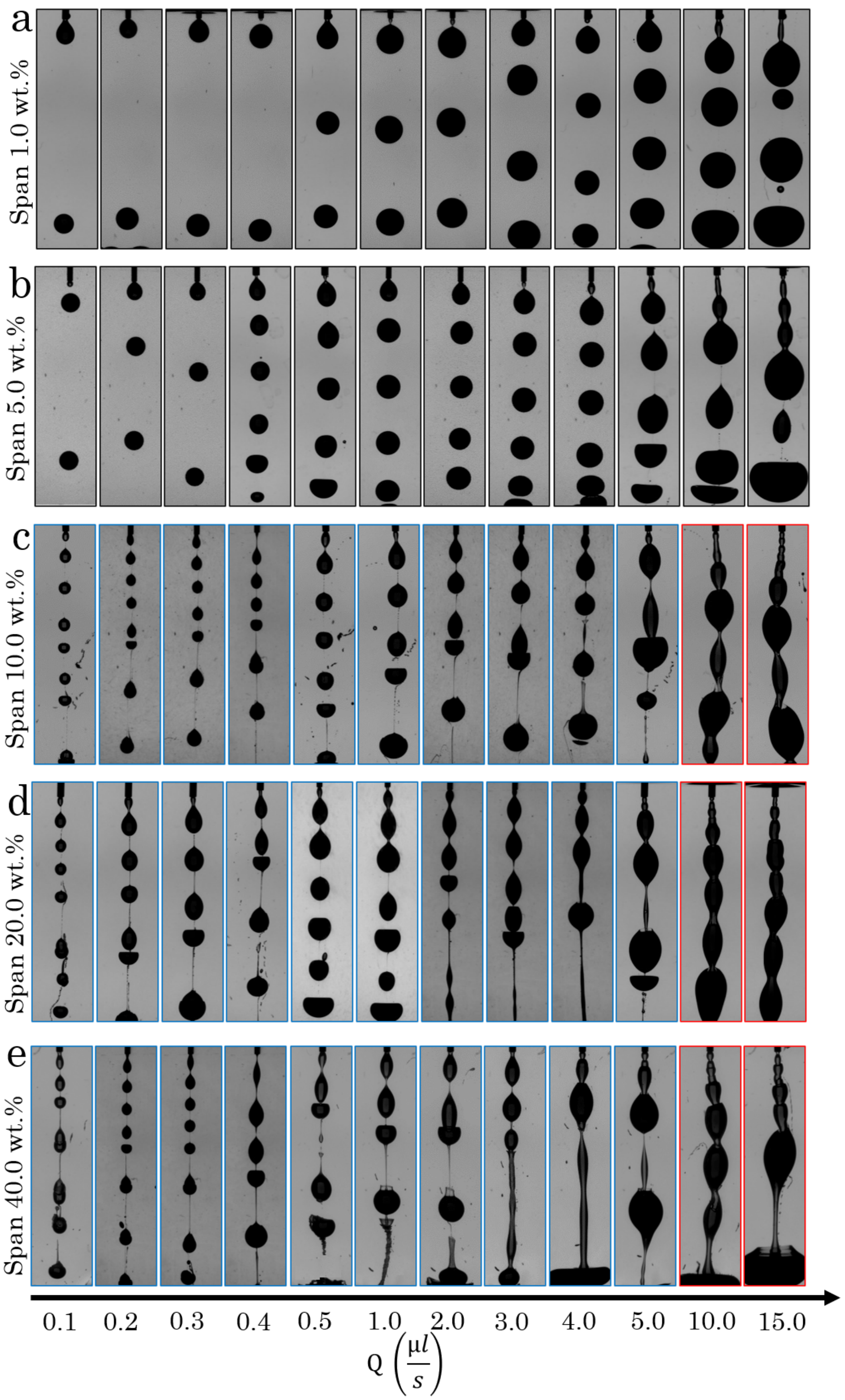

Figure S4: Injection of 1.0 wt.% silica into Span micellar solutions (Viscosity of mineral oil = 135 $mPa.s$) at concentrations of **(a)** 1.0 wt.%, **(b)** 5.0 wt.%, **(c)** 10.0 wt.%, **(d)** 20.0 wt.%, and **(e)** 40.0 wt.%. The diameter of the needle is 500 μm.

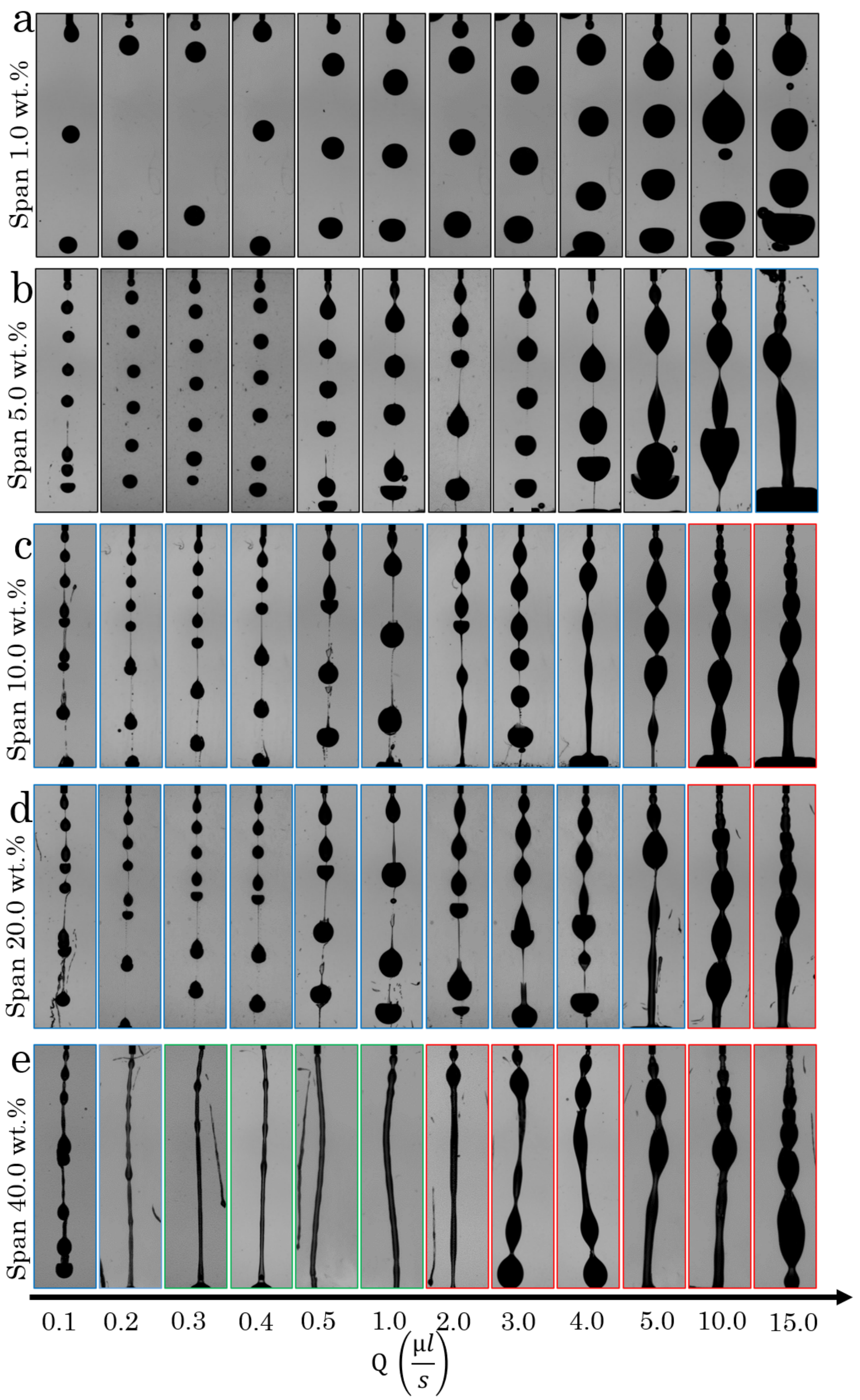

Figure S5: Injection of 2.0 wt.% silica into Span micellar solutions (Viscosity of mineral oil = 135 $mPa.s$) at concentrations of **(a)** 1.0 wt.%, **(b)** 5.0 wt.%, **(c)** 10.0 wt.%, **(d)** 20.0 wt.%, and **(e)** 40.0 wt.%. The diameter of the needle is 500 μm.

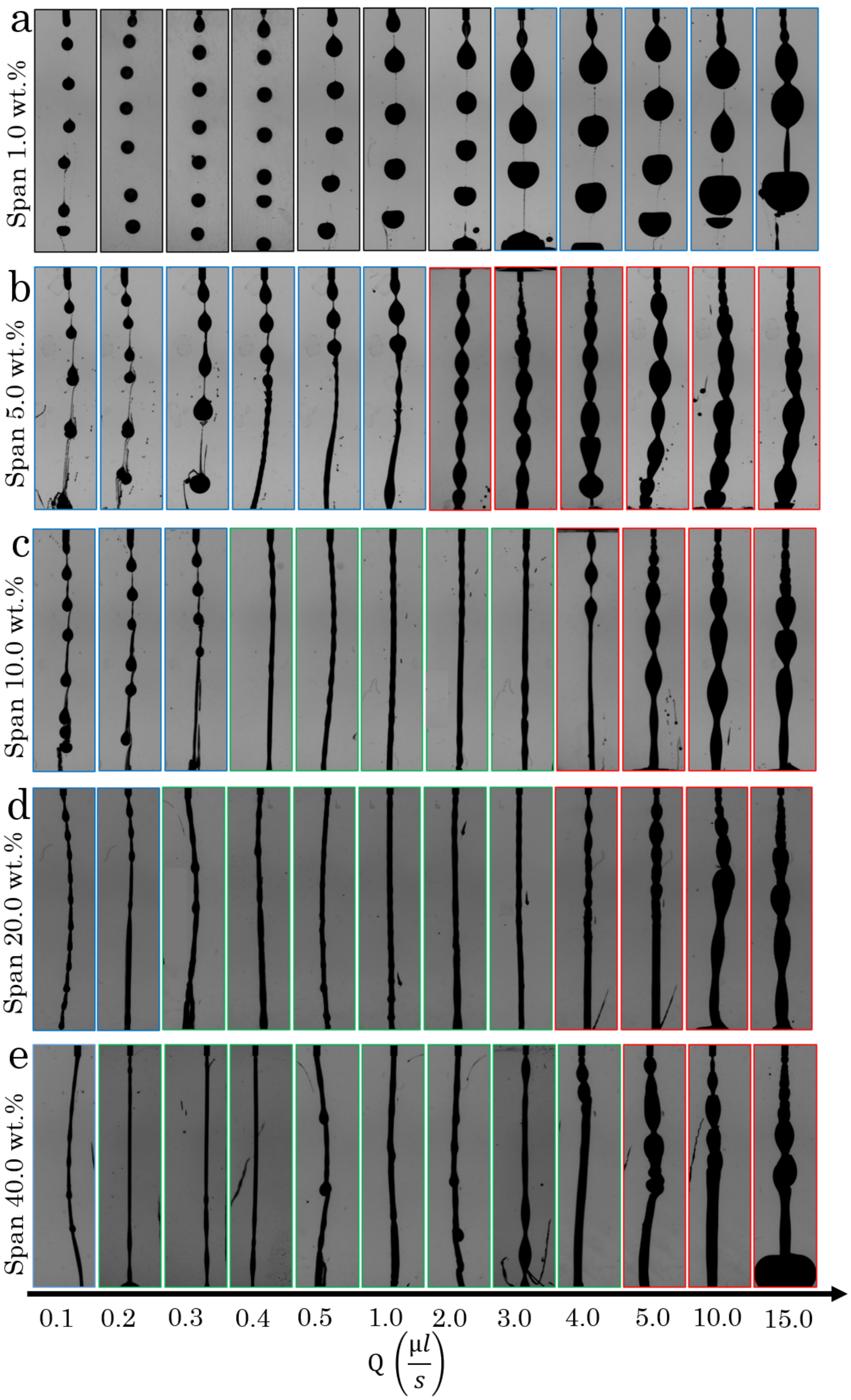

Figure S6: Injection of 4.0 wt.% silica into Span micellar solutions (Viscosity of mineral oil = 135 $mPa.s$) at concentrations of **(a)** 1.0 wt.%, **(b)** 5.0 wt.%, **(c)** 10.0 wt.%, **(d)** 20.0 wt.%, and **(e)** 40.0 wt.%. The diameter of the needle is 500 μm.

Supplementary Note 3: Fourier analysis

The flow regimes are identified based on the spectrum intensity of Fourier analysis. In brief, images are imported in Image J and binarized with an appropriate threshold value. The intensity amplitude over the length scale is then imported in Matlab where the spectrum intensity is calculated using the fast Fourier transform (FFT). The spectrum intensity is plotted over the inverse of wavelength and used for the analysis. **Fig. S7** summaries the image analysis procedure.

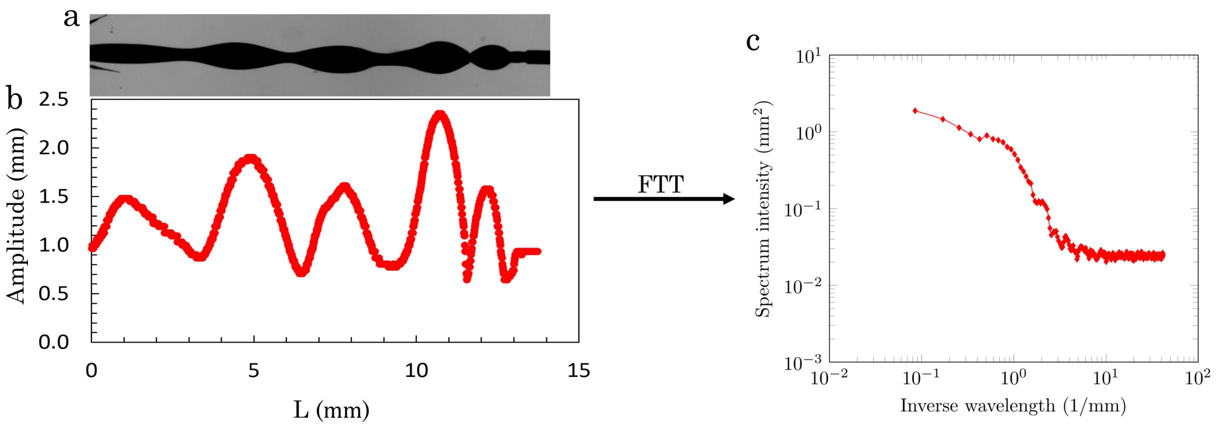

Figure S7: Image analysis. **(a)** The flow regime image, **(b)** The intensity over the flow length scale of the image, and **(c)** The spectrum intensity over over the inverse of wavelength. Source data are provided as a Source Data file.

FFT analysis is conducted over the range of tested particle and surfactant concentrations and injection speeds as presented in **Figs. S3-6**. Peaks in the power spectrum indicate the presence of a periodic pattern, while a monotonic power spectrum is expected for the data that lack periodicity. The power spectrum shows no characteristic length scale for column and connected regimes, while a peak with $\lambda_{\text{mean}} \sim 2$ mm is reported for the BOAS regime **Figs.**

S8a-c. Thus, we consider the cut-off wavelength, where the power spectrum starts decreasing, as evident for the characteristic wavelength in BOAS and connected flow regimes. **Figs. S8a-c** indicates that the spectrum intensity does not change considerably over time. Thus, images at t = 15 s from the start of the injection are used for the analysis. Time series images are provided in **Figs. S9-11**. **Fig. 1c** summarizes the mean and cutoff wavelength for the presented flow regimes in **Figs. S3-6**.

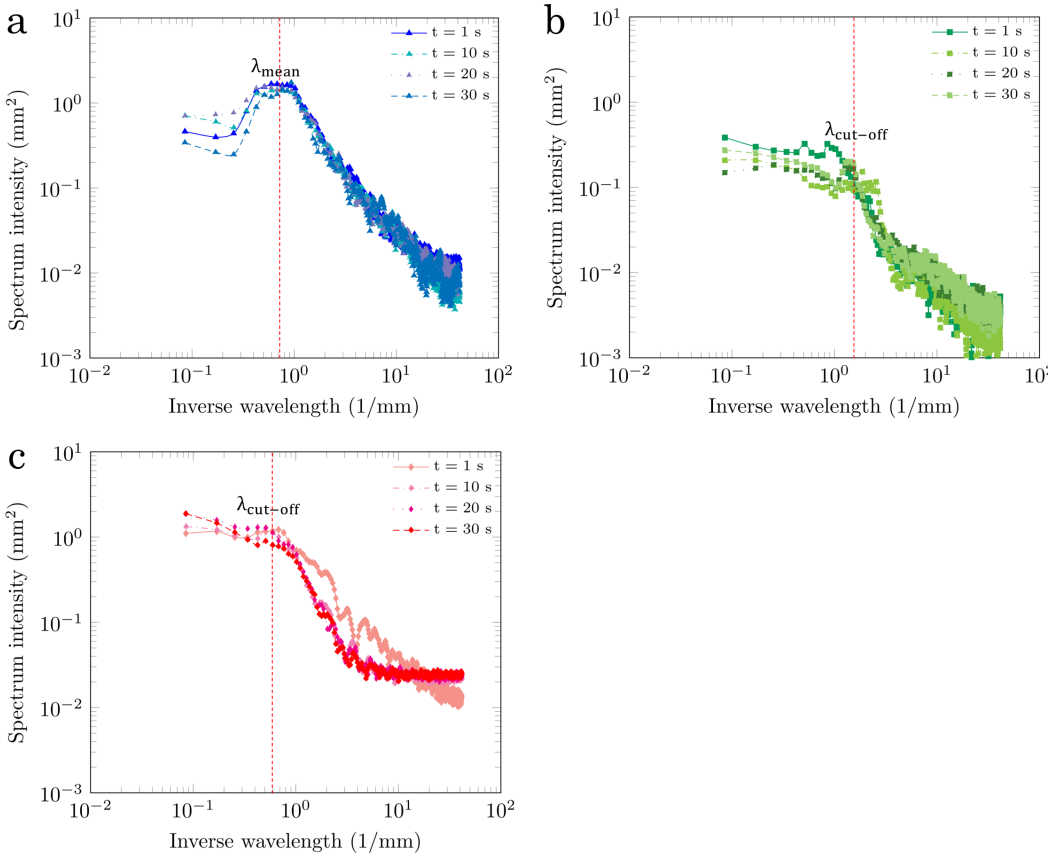

Figure S8: Fourier analysis. The spectrum intensity over inverse of wavelength **(a)** BOAS, **(b)** column, and **(c)** connected flow regimes. Source data are provided as a Source Data file.

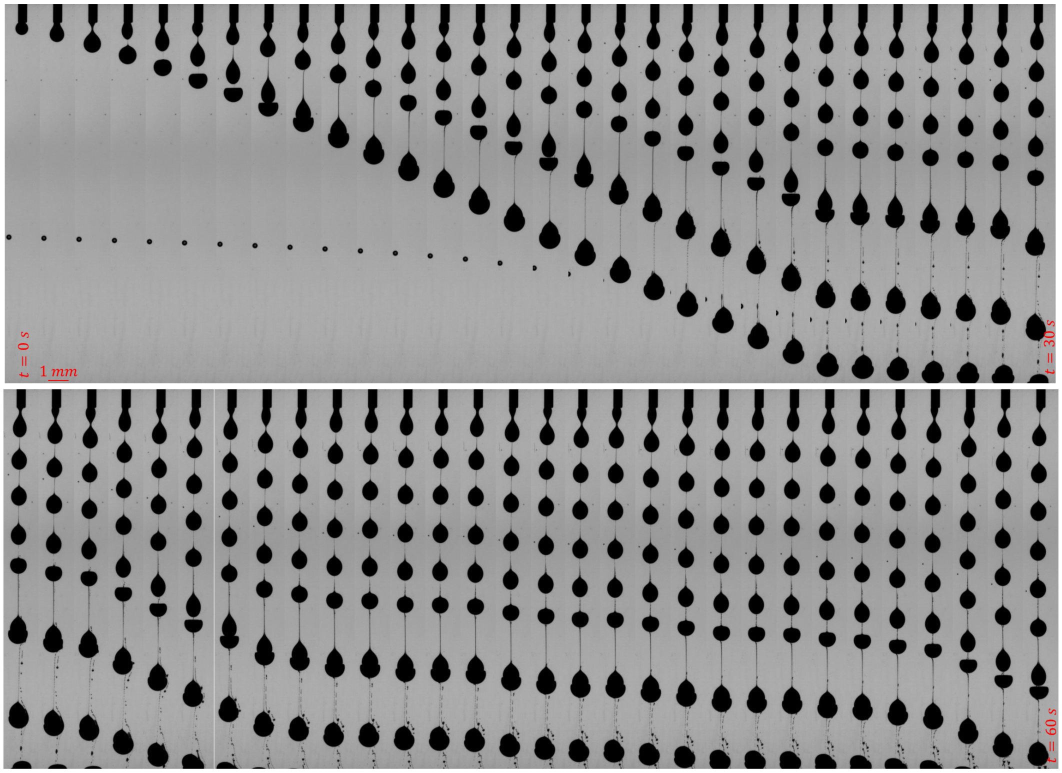

Figure S9: Time series images of the BOAS flow regime.

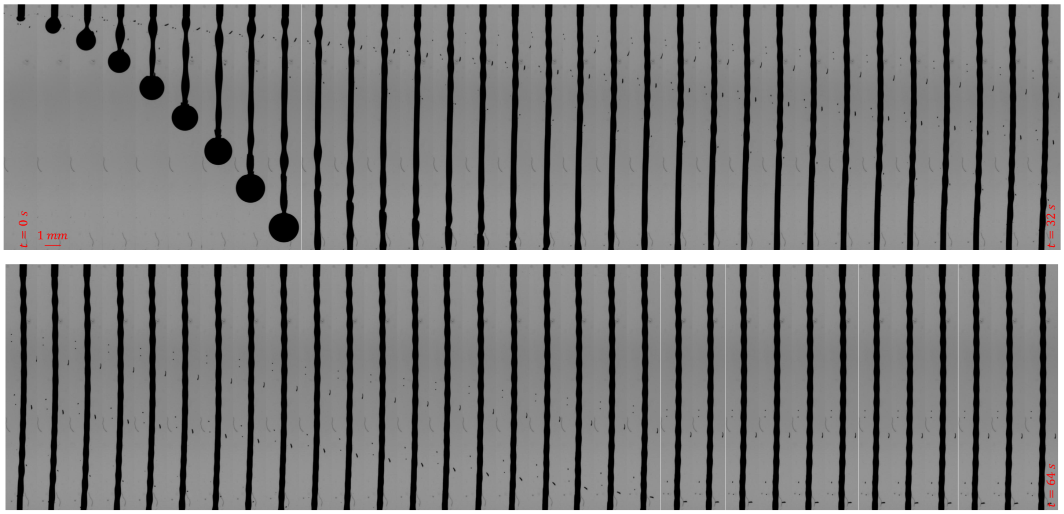

Figure S10: Time series images of the column flow regime.

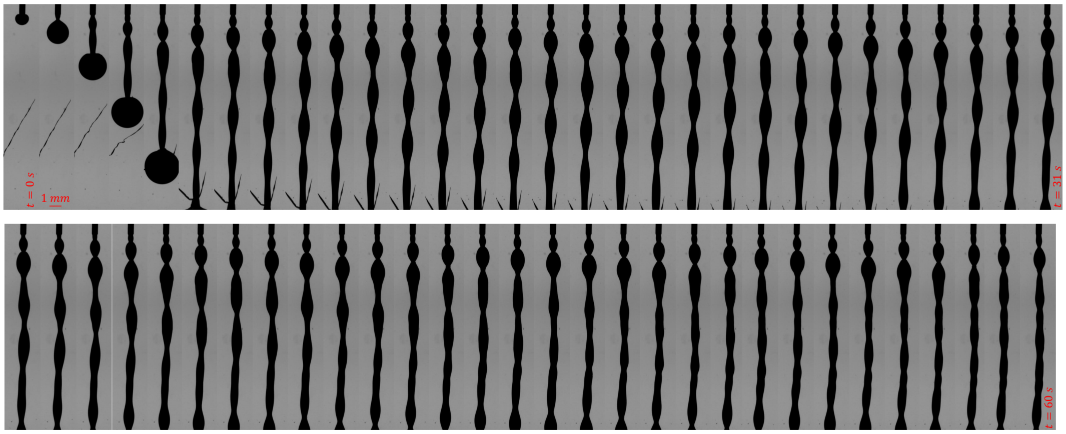

Figure S11: Time series images of the connected flow regime.

The 3D map of flow regimes, as shown in **Fig. 1d**, is reconstructed on the 2D planes of silica concentration-Span concentration, Weber number-silica concentration, and We number-Span concentration (**Fig. S12**).

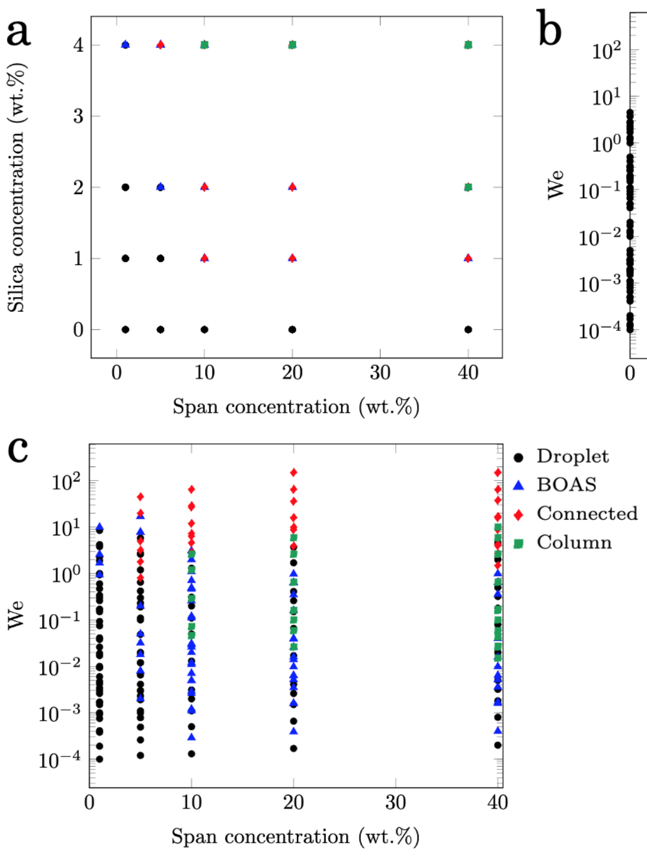

Figure S12: 2D planes of flow regime morphologies in Figure 1d. **(a)** silica concentration-Span concentration, **(b)** Weber number-silica concentration, and **(c)** We number-Span concentration. Source data are provided as a Source Data file.

Supplementary Note 4: Formation of interfacial materials in the static condition

3 ml of DI water is poured into a glass vial and 3 ml of 10 or 20.0 wt.% Span micellar solution is gently added at the top of the aqueous phase using a micro-pipette, **Fig. S13**. Initially, the oil phase is transparent. Upon contact, a shade of yellow color is observed in the micellar solution, indicating the diffusion of water into the oil phase and the possibility of the formation of an

emulsion phase [2]. Over time, a dark skin zone is observed at the glass wall, signaling that the glass is preferentially wetted by the emulsion. A similar wetting phenomenon has been reported previously in in-situ emulsification experiments with Span [2,3]. In the second experiment, a 4.0 wt.% silica nanoparticle dispersion is placed in contact with 20.0 wt.% Span micellar solution (**Fig. S13**). Unlike the DI water case, the oil phase becomes cloudy (yellowish) upon contact with the silica dispersion, where the color intensity grows over time. **Figure S14** shows the penetration of the aqueous phase (silica 4.0 wt.%) into the oil phase (Span 20.0 wt.%) that could be a contributing mechanism in the fast formation of emulsions in the presence of silica particles.

We collect samples from the oil-water interface of both cases after 24 hours of contact and take Cryo-SEM images in **Fig. S15**. **Figure S16** shows the average size of the emulsion droplet generated from different concentrations of silica particles.

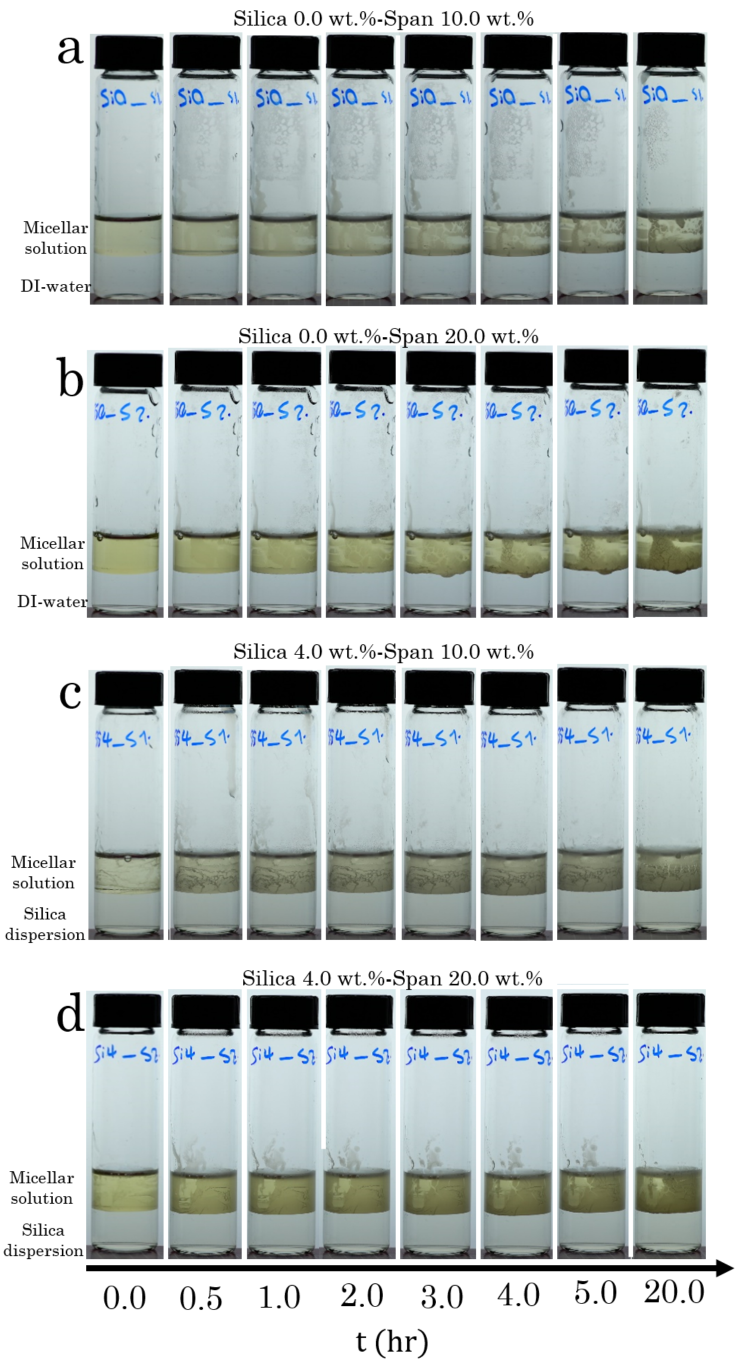

Figure S13: Injection of DI water into Span micellar solutions (viscosity of mineral oil = 135 $mPa.s$) at concentrations of **(a)** 1.0 wt.%, **(b)** 5.0 wt.%, **(c)** 10.0 wt.%, **(d)** 20.0 wt.%, and **(e)** 40.0 wt.%.

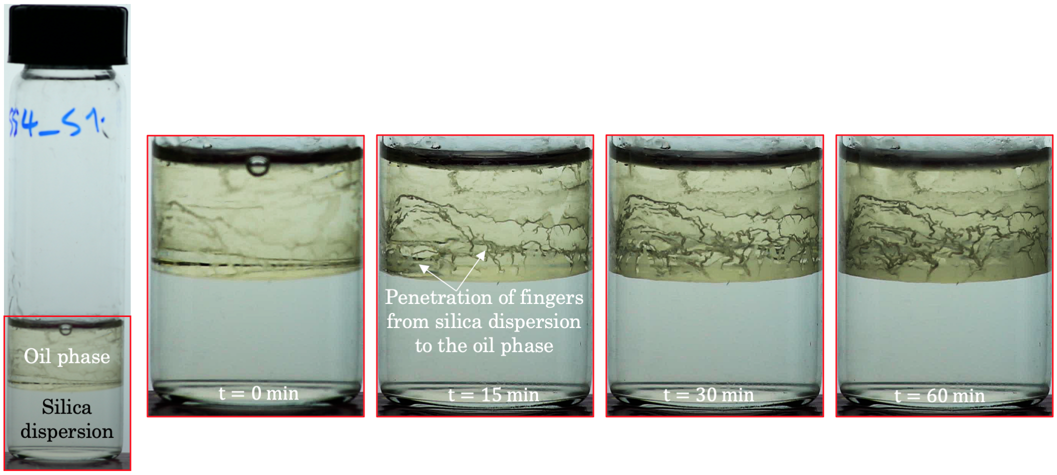

Figure S14: Expansion of the interfacial area and penetration of aqueous phase into the oil phase over time.

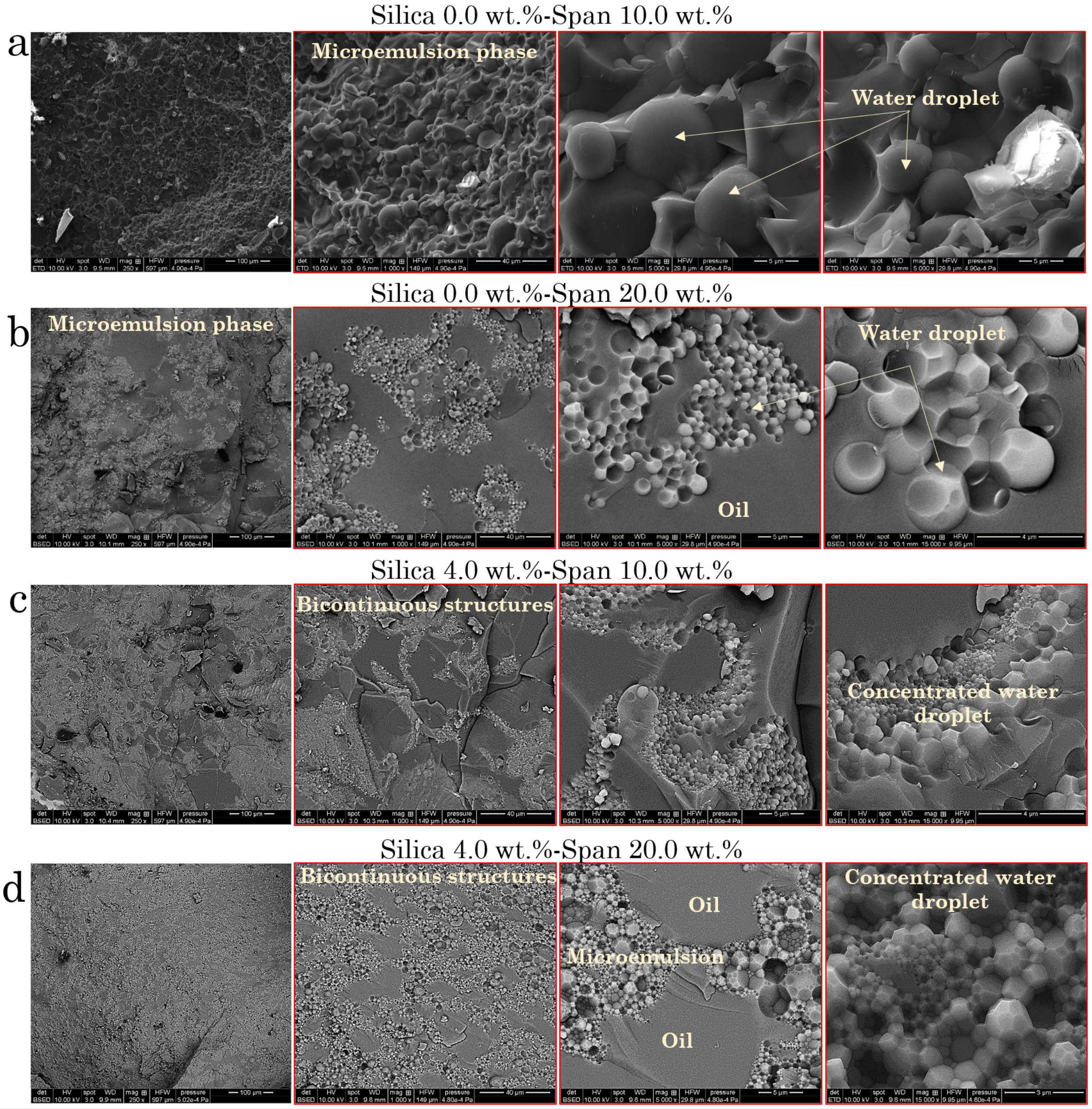

Figure S15: Cryo-SEM images of micellar solutions after contact with DI water and 4.0 wt.% silica dispersion. **(a)** DI water in contact with 10.0 wt.% Span micellar solution. **(b)** DI water in contact with 20.0 wt.% Span micellar solution. **(c)** 4.0 wt.% silica dispersion in contact with 10.0 wt.% Span micellar solution. **(d)** 4.0 wt.% silica dispersion in contact with 20.0 wt.% Span micellar solution. In Cryo-SEM images, the dark and light gray colors respectively represent oil and aqueous phases, while the nanoparticles are not visible.

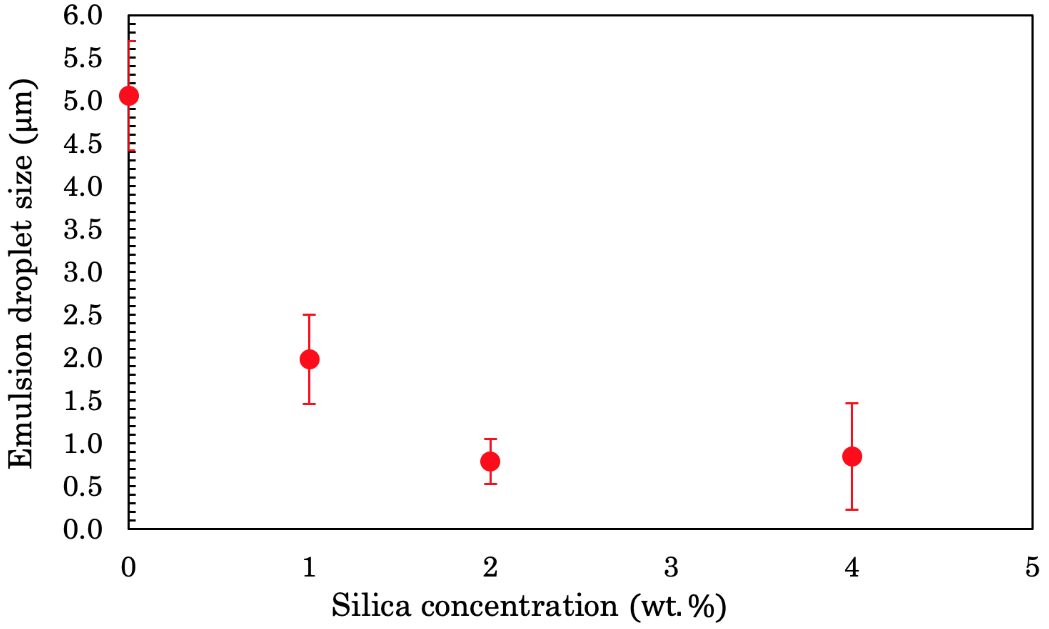

Figure S16: Emulsion droplet size (droplet diameter) over silica concentration. The droplet size is obtained from Cryo-SEM microscopic images. The error bars represent the standard deviation of droplet's size within the measurement region. Source data are provided as a Source Data file.

The high magnification confocal images of the interconnected structures of oil and water in **Fig. S17** shows the presence of small droplets of aqueous phase (red color) in the sample. Images are taken from a diluted region. Although there are many small sub-micrometer droplets within the sample, we cannot observe the submicrometric droplets due to the limitation in resolution.

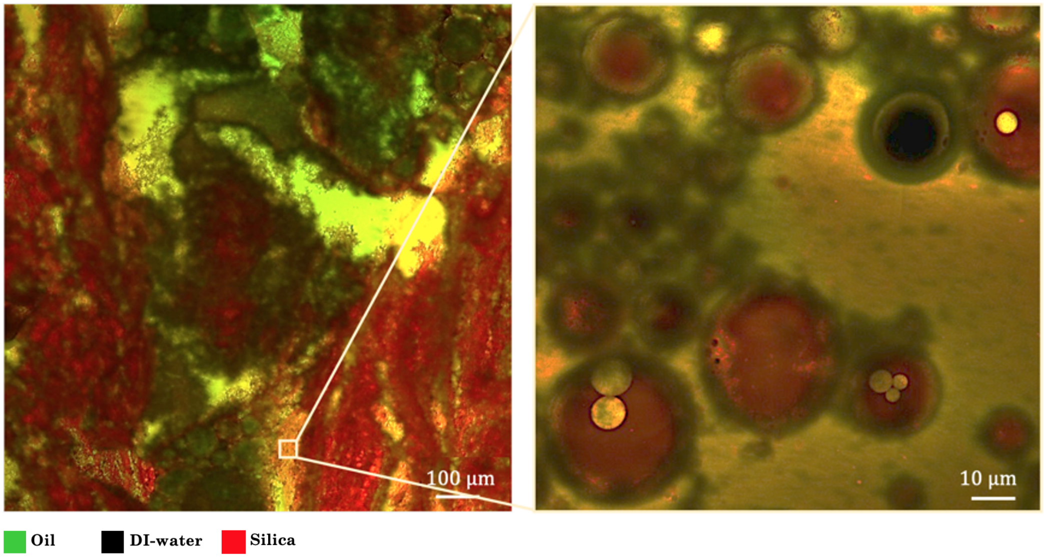

Figure S17: High magnification confocal images of the interconnected structures of oil and water that shows the presence of small droplets in the sample.

We quantify the rates of interfacial material formation, i.e., interconnected zones of emulsions and oil for the silica dispersion and emulsions for DI water. A droplet (V = 2 μl) of the aqueous phase is placed in the bulk of 20.0 wt.% Span micellar solution, close to a transparent solid surface as indicated schematically in **Fig. S18a**. Images are recorded from the bottom of the container over 24 hours. Note that the high concentration of Span micelles prevents the direct contact of the aqueous phase droplet with the bottom of the container. A film of oil is trapped within the micelles' chain, thus the droplet levitates over an oil cushion, and it is in a fully non-wetting condition. **Figs. 2a-b** depict that upon placing an aqueous phase droplet in Span micellar solution, the 4.0 wt.% silica droplet is flattened to twice the size of the DI water droplet. Also, the surface area of both droplets increases during the first 3-4 hours.

Please note that the generated interfacial materials on the surface of the DI water are not stable. We believe that the destabilization of the dark zone at the water-micellar solution

interface is a new phenomenon that has not been studied and discussed so far in literature. Since the focus of this manuscript is on the short time dynamics of the emulsion formation at silica dispersion-micellar solution interfaces that possibly lead to the liquid column formation, we did not probe the long time destabilization of the water-micellar solution interface, in the absence of silica antiparticles, as observed in **Figs. 2a-b**.

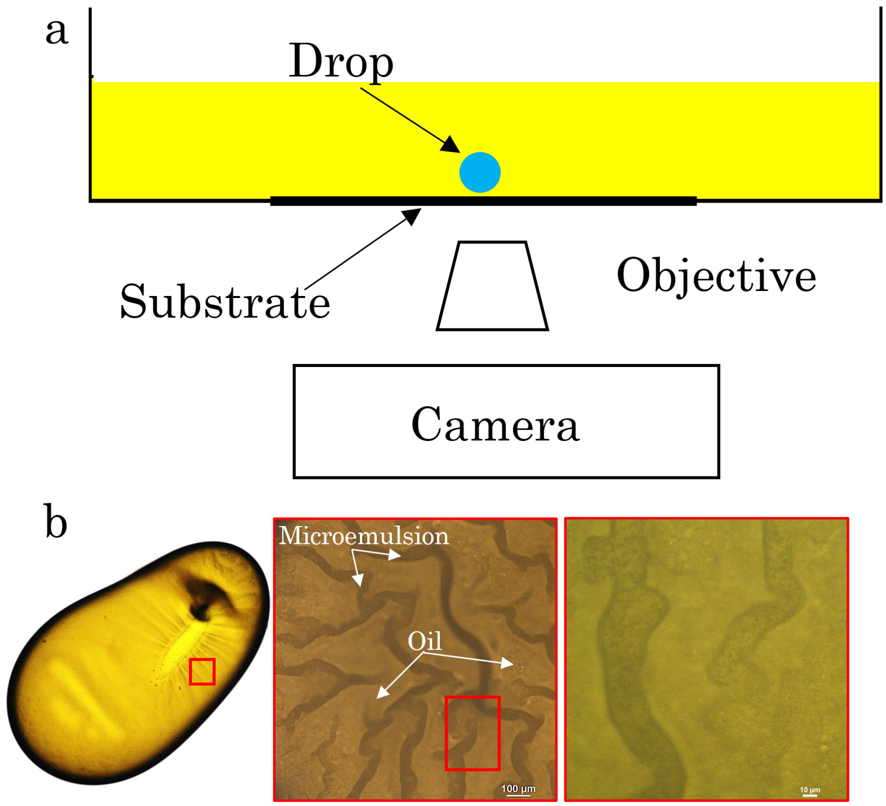

Figure S18: **(a)** Bottom view imaging the experimental set-up. A Petri dish is filled with the micellar solution and is placed on an inverted microscope. A droplet of aqueous phase (DI water or silica dispersion) is placed inside the micellar solution close to the solid surface. Images are recorded with the rate of 44 fps from the bottom view. **(b)** High magnification images of emulsions.

Supplementary Note 5: Rheological properties of emulsions

We collected samples from the interface of silica 2.0 and 4.0 wt.% after the contact with various Span concentrations (1.0 to 40.0 wt.%), and conducted oscillatory shear experiments, i.e., frequency sweep test at the fixed strain of 0.2 % and presented the results in **Fig. S19**. For the samples collected from the interface of 2.0 wt.% silica dispersion, both elastic and viscous moduli increase by increasing the Span concentration and the angular frequencies. The sample generated from silica 2.0 wt.%-Span 40.0%, which can form a stable liquid filament, has the largest elastic and viscous moduli at the highest frequencies (**Fig. S19a**). Generated samples at silica 2.0 wt.% for all tested Span concentration interfaces, silica 4.0 wt.%-Span 1.0 wt.%, and silica 4.0 wt.%-Span 5.0 wt.%, have a cross-over point where at low frequencies the elastic modulus is higher than the viscous modulus. The generated samples at silica 4.0 wt.%-Span 10.0, 20.0, and 40.0 wt.% interfaces have an elastic modulus greater than 100 Pa, which is always greater than or equal to the viscous modulus. Also, the elastic modulus for these cases reaches a plateau at the low frequencies, an indicator of gel-like materials [4]. For the two highest concentrations (silica 4.0 wt.%-Span 20.0 and 40.0 wt.%), the elastic and viscous moduli have close values indicating that the materials have a gel-like behaviour. Similar viscoelastic responses have been reported for the Bijles formed from jamming of nanoparticle-surfactants at the oil-water interfaces [5].

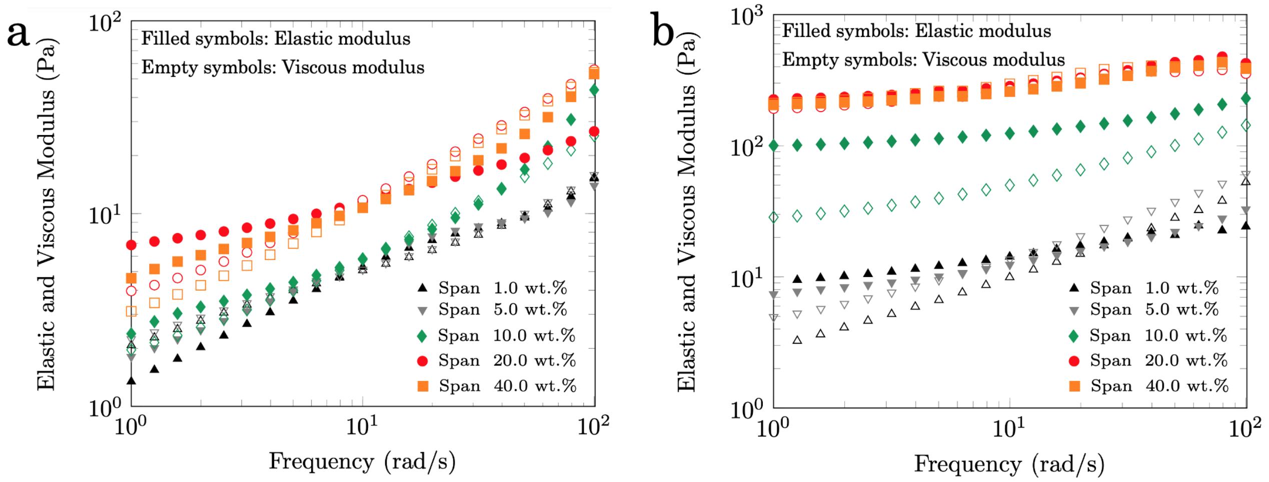

Figure S19: Rheological properties of the generated interfacial material at the aqueous phase-Span micellar solution interfaces. **(a)** Silica 2.0 wt.% and **(b)** silica 4.0 wt.%. The filled and empty symbols represent the elastic and viscous moduli, respectively. Source data are provided as a Source Data file.

Supplementary Note 6: The effect of surrounding oil viscosity on the formation of liquid columns

We study the effect of the surrounding liquid viscosity by using two other oil samples with viscosities 30 mPa.s and 1000 mPa.s. **Fig. S20** presents the results of the injection of 4.0 wt.% silica dispersion into the 20.0 wt.% Span micellar solution with the three oil viscosities 30 mPa.s in **Fig. S20a**, 135 mPa.s in **Fig. S20b**, and 1000 mPa.s in **Fig. S20c**, respectively. Liquid columns are not generated at any of the examined injection flow rates in low viscosity oil (μ = 30 mPa.s). Increasing the viscosity to 1000 mPa.s results in the formation of liquid columns for all injection flow rates studied. We inspect the oil-silica dispersion interface using

confocal microscopy as shown in the second column of **Figs. S20a-c**. In the confocal images, green, black, and red colors represent oil, water, and silica particles, respectively. The images reveal that the spontaneously generated interfacial layer in the low viscosity oil system is similar to the multiple emulsions as previously reported with heptane [6]. The interconnected structures of oil an emulsions are formed only at 135 mPa.s and 1000 mPa.s oil interfaces.

In the low viscosity oil (μ = 30 mPa.s), γ continuously increases over time (**Fig. S20d**), indicating the rapid formation of emulsion at the interface and their subsequent detachment from the interface [6]. However, in two higher viscosity micellar solutions, γ has an initially sharp decrease, reaching a plateau in 200 seconds. The constant γ in these systems indicates that the generated emulsion phase remains at the interface, forming a highly concentrated emulsion zone (**Fig. S21**).

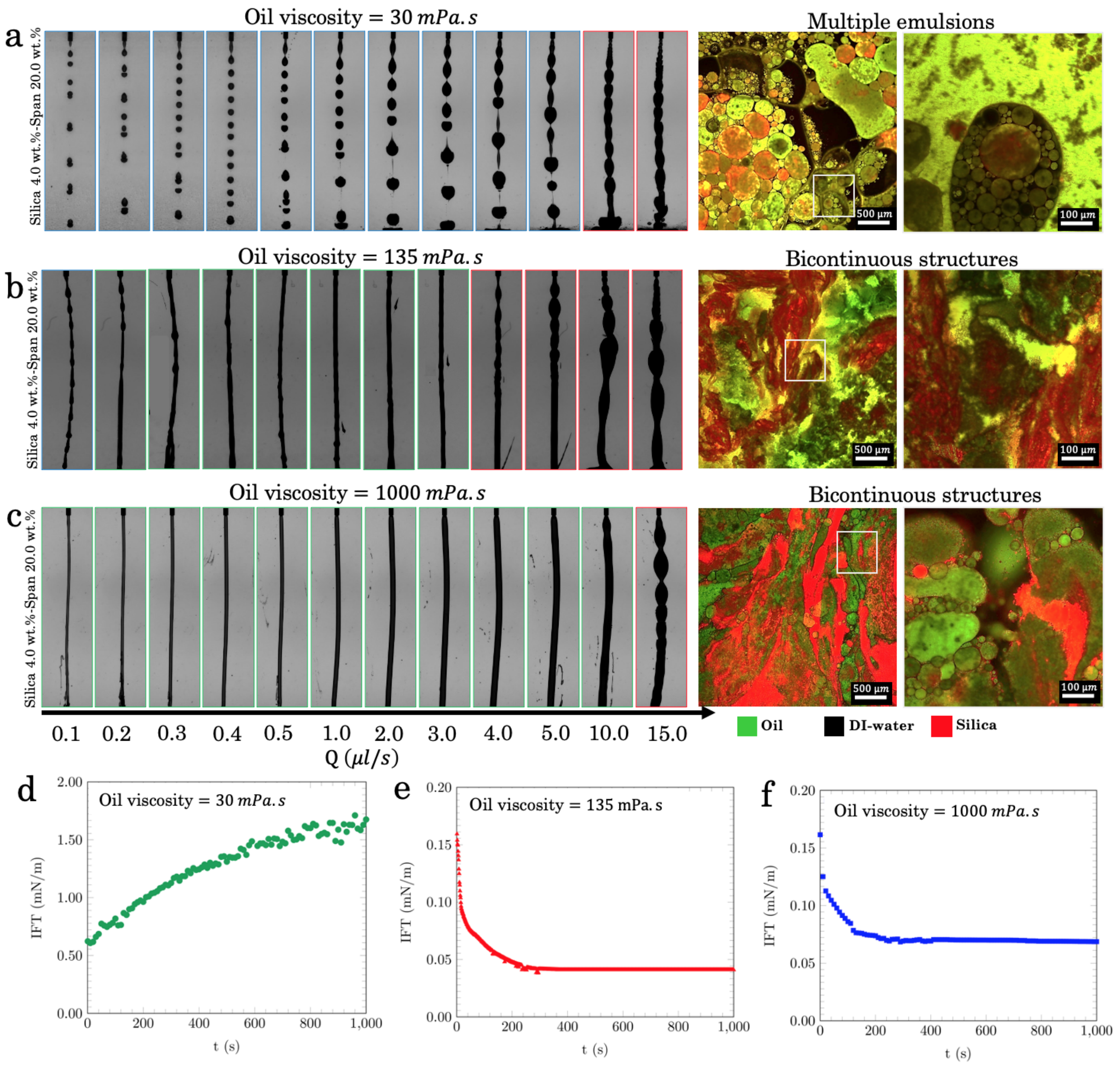

Figure S20: Effect of surrounding liquid viscosity on the formation of liquid columns and emulsion-based interfacial layer. 4.0 wt.% silica dispersion is injected into 20.0 wt.% Span micellar solution made of oil with the viscosity **(a)** 30 mPa.s to **(b)** 135 mPa.s, and **(c)** 1000 mPa.s. Microscopic images of oil-water interface of each case are shown on the right side. Interfacial tension of 4.0 wt.% silica dispersion -micellar solution with the viscosity of **(d)** 30 mPa.s, **(e)** 135 mPa.s, and **(f)** 1000 mPa.s. Source data are provided as a Source Data file.

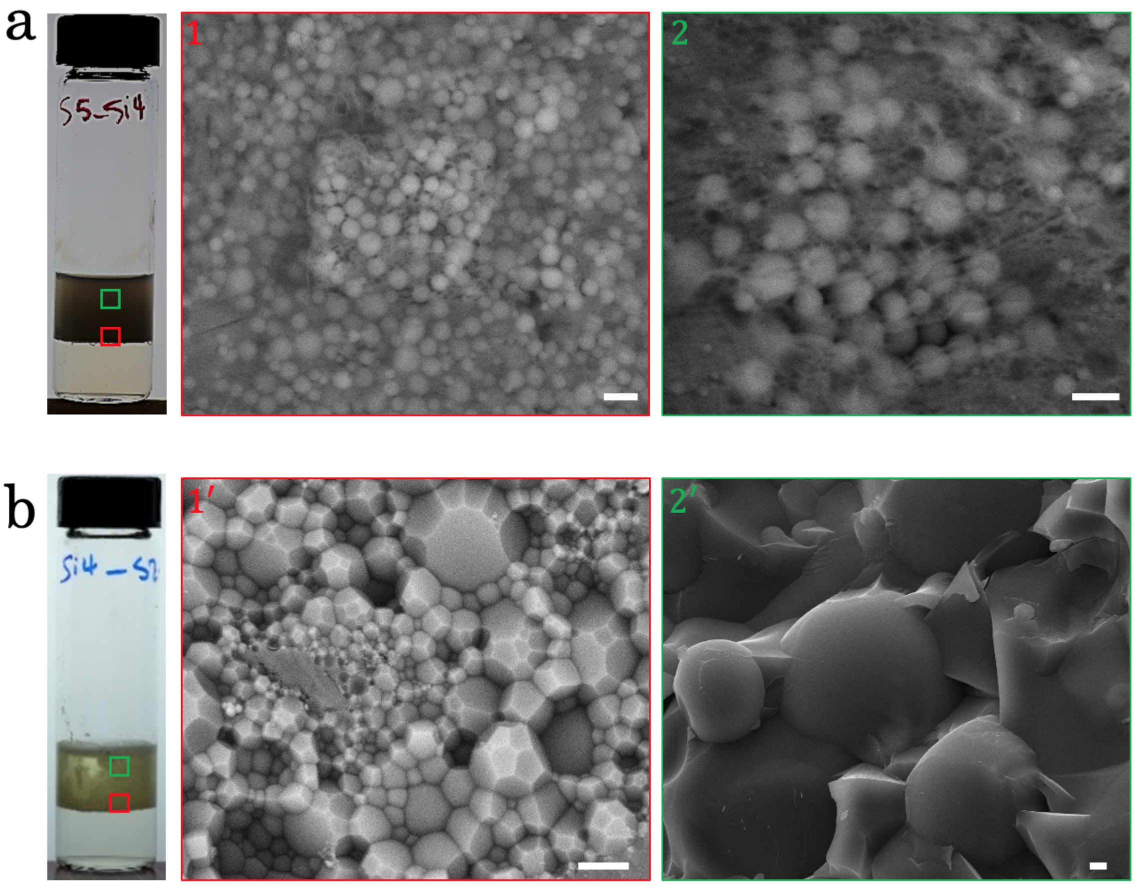

Figure S21: Cryo-SEM images of emulsion zone in low viscous and high viscous oil systems. (**a**) Heptane ($\mu = 0.3$ mPa.s) and (**b**) Mineral oil ($\mu = 135$ mPa.s). The scale bar represents 1 μm.

Supplementary Note 7: Dimensional analysis

Rayleigh-Plateau instability analysis

We calculate the break-up time of the liquid filament in the presence and absence of nanoparticles with the assumption that no interfacial materials are formed at the water-oil interface. So, only the interfacial tension and fluids' viscosities are the contributing factor in the filament

instability. For a liquid filament (radius of R, viscosity of μ_i) flowing through a fluid with the viscosity of μ_e in a container with the width of W, the rate of disturbance grow is [7,8]:

$$\omega = \left(\frac{\gamma}{16\mu_\mathrm{e}\mathrm{W}}\right)\left[\frac{\mathrm{F}(\mathrm{x},\lambda)(\mathrm{k}^2-\mathrm{k}^4)}{\mathrm{x}^9(1-\lambda^{-1})-\mathrm{x}^5}\right] \qquad \text{(Eq. S.1)}$$

k is the dimensionless wavenumber of the perturbation and it is considered as 0.257 based on the viscosity ratio $\mu_\mathrm{i}/\mu_\mathrm{e} = \mathrm{O}(0.001)$ [9], x is the dimensionless radius of the thread ($\mathrm{x} = \mathrm{R}/\mathrm{W}$), λ is the viscosity ratio $\mu_\mathrm{i}/\mu_\mathrm{e}$, and $\mathrm{F}(\mathrm{x},\lambda) = \mathrm{x}^4(4-\lambda^{-1}+4\mathrm{lnx}) + \mathrm{x}^6(-8+4\lambda^{-1}) + x^8(4-3\lambda^{-1}-(4-4\lambda^{-1})lnx)$ [7, 8]. In the absence of nanoparticles (DI water), the equation predicts a maximum growth rate of $\mathrm{w}(\mathrm{k}=0.257) = 61\ \mathrm{s}^{-1}$. Assuming that perturbations at the entrance initially are of nanometer size and perturb the radius as $\mathrm{r} = \mathrm{r}_0 + \epsilon_0 e^{i(k/r_0)z+wt}$ until $\mathrm{r}_0 = \epsilon_0 e^{wt}$, the break-up time is predicted as $\mathrm{t} = \omega^{-1}\ln(200\times10^3) = 0.2$ s. In the presence of silica particles (in systems that the liquid filament is generated as an example Span 20.0 wt.%-Silica 4.0 wt.%), $\mathrm{w}(\mathrm{k}=0.257) = 2.5\ \mathrm{s}^{-1}$, the predicted break-up time is t = 4.9 s. Unlike the systems without nanopartciles, the calculated instability time in the presence of nanoparticles does not match the flow regime transition in **Fig. S6**. Thus, we concluded the low interfacial tension of the system, and the high viscosity of the surrounding phase cannot be the only factors in stabilizing liquid filaments in our system.

Diffusion and emulsification time scales

Flow regime transitions from BOAS to column and from column to connected are observed for a series of experiments as depicted in **Fig. S22**, where the first transition determines the diffusion time and the latter one represents the emulsification time, as summarized in **Table**

S1.

For the sets of experiments that the BOAS to column flow regime transition occurs (**Fig. S22** and **Table S1**), the diffusion time is a linear function of micellar solution viscosity (μ), consistent with the Stokes-Einstein theory of diffusion in solution (**Fig. 3b**). Thus, in experiments where BOAS to column transition does not occur within the time frame of experiment (0.2-18.0 seconds), we calculate the diffusion time as $\mathrm{t_D} \simeq \ell^2/\mathrm{D} \simeq (6\pi \mathrm{a}\ell^2/\mathrm{k_B T})\mu$, where a, ℓ, k_B, and T are, respectively, the micelle diameter, diffusion length scale, Boltzmann constant, and temperature. A characteristic length (ℓ) of 1 μm perfectly fits the linear relationship of $\mathrm{t_D} \sim \mu$, as presented in **Fig. 3b**. It is worth mentioning that the distance between two micelles in the bulk oil is close to 1 μm based on the cryo-SEM images in **Fig. S21b**, panel $2'$.

The experimental evaluation of the transition time from column to connected flow regimes reveals that the emulsification time is a linear function of the equilibrium interfacial tension in systems with $\gamma_{\mathrm{eq}} \geq 0.06$ mN/m. In systems where the column to connected flow regime transition does not occur within the time frame of experiment, the emulsification time is calculated as $\mathrm{t_E} = \mathrm{C}\gamma_{\mathrm{eq}}$, where $\mathrm{C} = 1.12 \times 10^4$ $\mathrm{s^3/kg}$ is the constant determined from the experimental data presented in **Fig. 3b**. C is a function of change in surface area (required for the formation of a new interface) and the work required to increase the interface and can be expressed in terms of $\mathrm{C} = \mathrm{a}^2/(\kappa \mathrm{k_B T})$, where a, κ, k_B, and T are, respectively, the micelle diameter, the emulsification kinetic rate constant, Boltzmann constant, and temperature. Considering the initial micellar size of 5 nm and the ambient condition in the above equation, κ is 0.54 $\mathrm{s^{-1}}$. As expected, this rate is slightly higher than the kinetic rates of 0.05-0.1 $\mathrm{s^{-1}}$ for silica nanoparticle-Span micellar systems at lower surfactant concentrations [6].

It should be mentioned that we also attempted to calculate the emulsification time and kinetic rates from the dynamic interfacial tension data obtained using the spinning drop method (SDT). In the presence of silica particles, the interfacial tension of micellar solutions significantly decreases from $3 - 6$ mN/m to values less than 1 mN/m. However, due to a 1-10 second lag-time between placing the measurement chamber in the SDT holder and the start of recording the interfacial tension data, we lose the major reduction in interfacial tension, which is expected to be an exponential function of time. Thus, the recorded dynamic interfacial tension data cannot fully represent the emulsification rate. Nevertheless, the initial assessment of flow regime images in **Fig. S3-6** reveals that the emulsification time is a function of equilibrium interfacial tension.

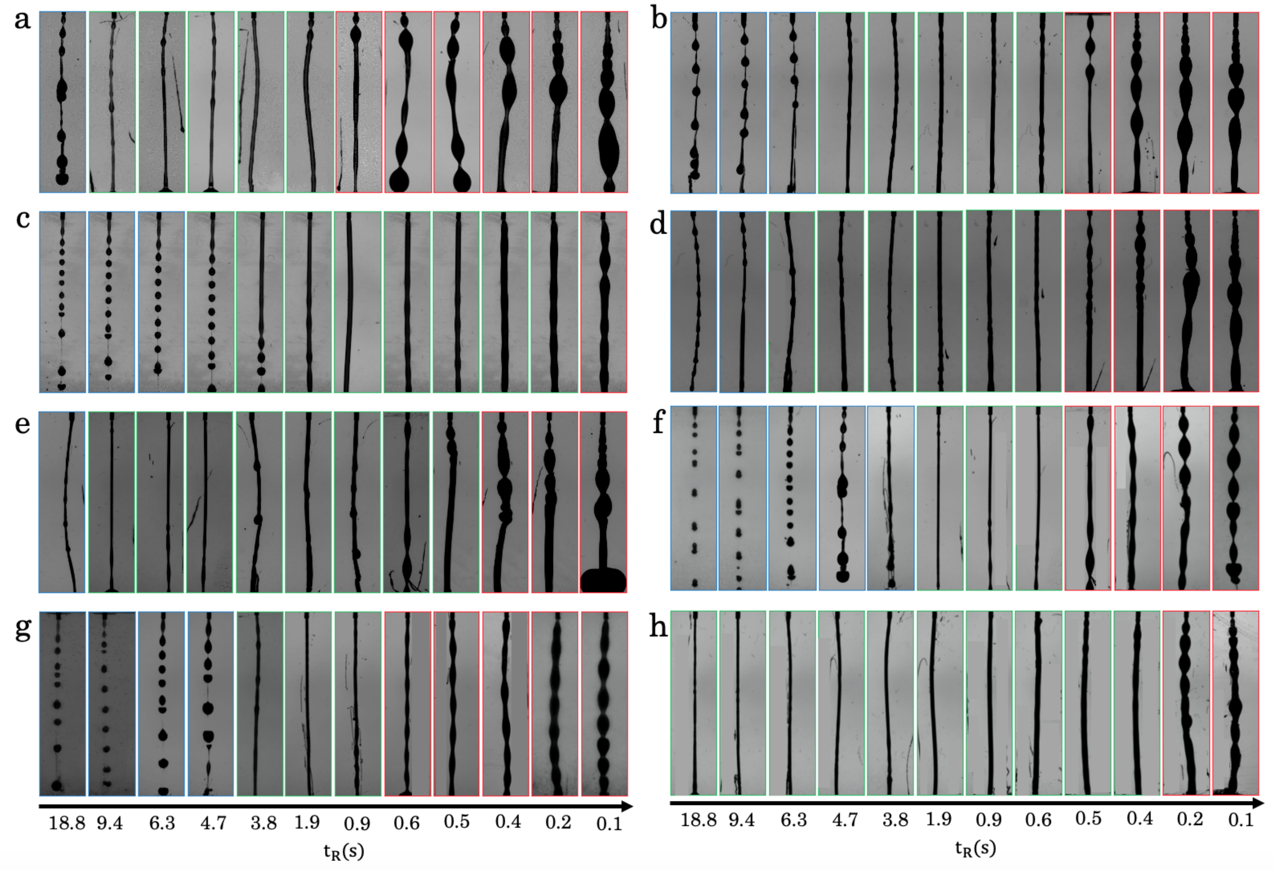

Figure S22: Flow regime transition, and emulsification-diffusion time scales. (**a**) Span 40.0 wt.% ($\mu = 260$ mPa.s)-silica 2.0 wt.% ($\mu = 1.1$ mPa.s), (**b**) Span 10.0 wt.% ($\mu = 180$ mPa.s)-silica 4.0 wt.% ($\mu = 1.2$ mPa.s), (**c**) Span 10.0 wt.% ($\mu = 180$ mPa.s)-silica 4.0 wt.% ($\mu = 4.4$ mPa.s), (**d**) Span 20.0 wt.% ($\mu = 220$ mPa.s)-silica 4.0 wt.% ($\mu = 1.2$ mPa.s), (**e**) Span 40.0 wt.% ($\mu = 260$ mPa.s)-silica 4.0 wt.% ($\mu = 1.2$ mPa.s), (**f**) Span 10.0 wt.% ($\mu = 63$ mPa.s)-silica 4.0 wt.% ($\mu = 1.2$ mPa.s), (**g**) Span 10.0 wt.% ($\mu = 120$ mPa.s)-silica 4.0 wt.% ($\mu = 1.2$ mPa.s), (**h**) Span 10.0 wt.% ($\mu = 480$ mPa.s)-silica 4.0 wt.% ($\mu = 1.2$ mPa.s).

Table S1: Flow regime transition times of the presented experiments in **Figure S22**.

Silica-Span systems	μ_{in}(mPa.s)	μ_{out}(mPa.s)	γ_{eq}(mN/m)	$t_D(s)$	$t_E(s)$
**a**	1.1	260	0.15 ± 0.03	9.4	1.9
**b**	1.2	180	0.09 ± 0.02	4.7	0.6
**c**	4.4	180	0.05 ± 0.02	4.7	0.2
**d**	1.2	220	0.04 ± 0.01	6.3	0.5
**e**	1.2	260	0.04 ± 0.01	9.4	0.5
**f**	1.2	63	0.05 ± 0.01	1.9	0.6
**g**	1.2	120	0.10 ± 0.02	3.8	0.9
**h**	1.2	480	0.05 ± 0.02	[redacted]	0.4

Properties of the inner fluid

The Reynolds number ($\mathrm{Re} = \rho_\mathrm{i}\mathrm{U}\mathrm{d}_\mathrm{i}/\mu_\mathrm{i}$, where ρ_i, μ_i, U, d_i are density and viscosity of the aqueous phase, injection speed, and inner diameter of the needle, respectively) is plotted as a function of Capillary number ($\mathrm{Ca} = \mathrm{U}\mu_\mathrm{i}/\gamma$, where μ, U, and γ are viscosity of the aqueous phase, injection speed, and IFT, respectively) in **Fig. S23**. Data points fall in parallel lines with the slope of ~ 1, indicating that a ratio of Capillary to Reynolds numbers can represent the parameters that influence the set of experiment. Thus, we utilize $\mathrm{Re}/\mathrm{Ca} = \mathrm{Oh}^2$ (where $\mathrm{Oh} = \mu_i/\sqrt{\rho_i \gamma d_i}$ is the Ohnesorge number; $\mathrm{Oh} = \mu_i/\sqrt{\rho_i \gamma d_i}$, μ_i and ρ_i, are the viscosity and density of the inner fluid and d_i is the inner diameter of the needle) along with convection and diffusion Damköhler numbers ($\mathrm{Da_C}$, $\mathrm{Da_D}$) to create a 3D phase diagram in **Fig. 3d** that distinguishes four flow regimes observed in **Fig. 1** and **Figs. S3-6**.

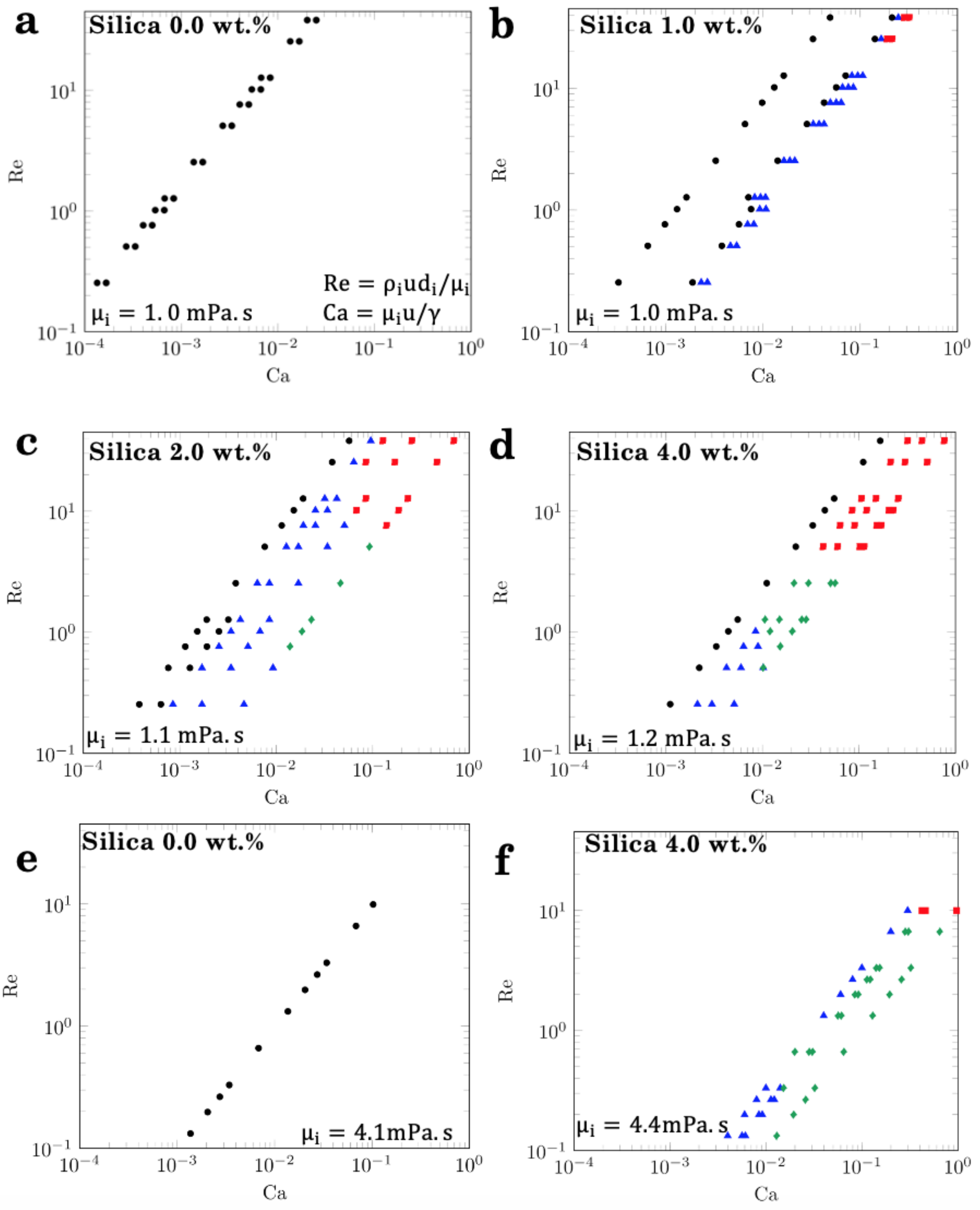

Figure S23: Reynolds and Capillary numbers of the flow regimes in **Figs. S2-5** and two sets of additional experiments with higher inner fluid viscosity. Source data are provided as a Source Data file.

Supplementary Note 8:Stability of the printed textures

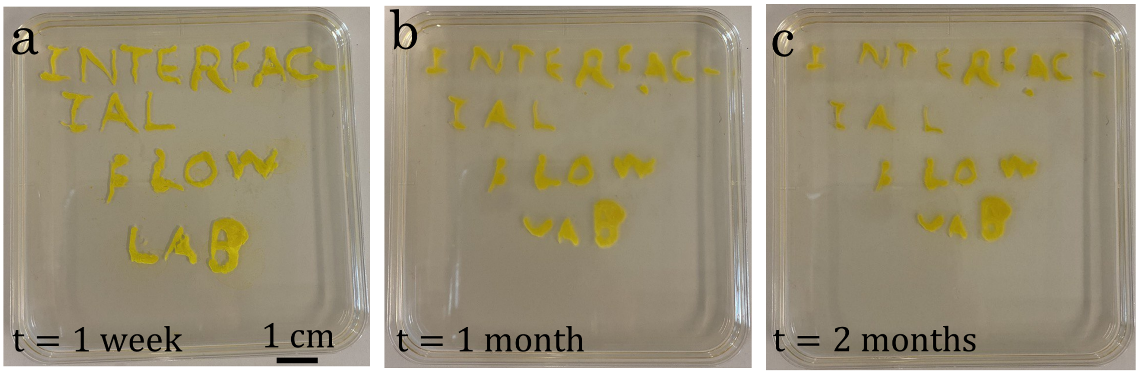

Figure S24: Stability of the printed liquid in liquid structures over time.

Supplementary Note 9: 3D images of the emulsification process

Figure 25a shows a DI water droplet that is placed in the bulk of mineral oil (0.0 wt.% Span concentration) close to the solid surface. As expected, the droplet spreads over the solid and forms a semi-spherical shape, minimizing the surface area with the surrounding liquid. Once oil is replaced with 20.0 wt.% Span micellar solution in **Fig. 25b**, the DI water droplet forms a disk with the height of $\sim 400\ \mu m$, as opposed to the equilibrium semi-spherical shape of a water droplet in clean mineral oil. Replacing the DI water droplet with 4.0 wt.% silica droplet in **Fig. 25c** shows that silica droplet covers a wider surface area, and its thickness is $\sim 200\ \mu m$. The capillary length, $\lambda_c = (\gamma/\triangle\rho g)^{1/2}$ (where γ is the interfacial tension, $\triangle\rho$ is the density difference between oil and aqueous phases, and g is the gravitational acceleration), of DI water in mineral oil is $\sim\ 6\ mm$, thus the shape of the DI water droplet ($d = 1.5\ mm$) in **Fig. 25a** is dominated by surface tension forces. On the other hand, the capillary lengths for DI water and

silica droplets in the Span micellar solution are $\sim$ 1 mm and $\sim$ 0.3 mm, respectively. Since the diameter of droplets is larger than the capillary length in these two cases (**Figs. 25b-c**), the shape of the droplet is controlled by gravity.

Wrinkles are generated on the surface of the silica droplet. A higher magnification image of **Fig. 25c** reveals that wrinkles are interconnected structures of oil and emulsions similar to the observed structures in Cryo-SEM images. **Fig. 25d** shows a slice (1/4) of silica dispersion droplet (**Fig. 25c**) at three different time steps (5, 15, and 30 minutes). Initially, the mixed green-black zones (emulsion phase) are only formed at the top and bottom of the droplet, which are in direct contact with the micellar solution. Over time the thickness of the emulsion phase increases, indicating that the emulsification advances in the depth of the droplet. We analyze the z-stack of images in **Figs. 25b-c** and calculate the growth of emulsification into the DI water droplet (**Fig. S26**) and 4.0 wt.% silica dispersion droplet (**Fig. S21**) in 20.0 wt.% Span micellar solution. In the DI water case, the emulsification advances $\sim$ 20 μm from the bottom and top of the droplet toward the center in the first time interval of 20 minutes and it does not reach the center of DI water droplet within an hour, **Fig. S26**. In the silica droplet, the emulsion phase grows $\sim$ 50 μm from the bottom and top of the droplet toward the center in the first time interval, **Fig. S27** and reaches the middle layer within one hour. Unlike the DI water droplet, the intensity of the emulsion phase does not decrease over time in the case of a silica drop.

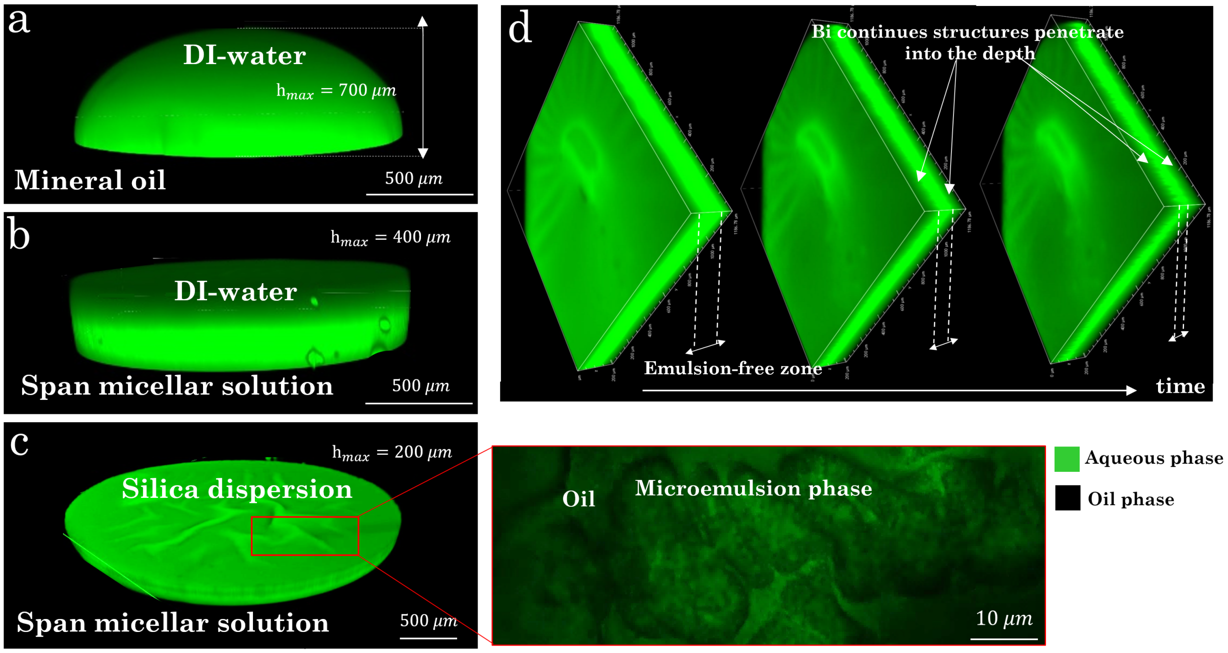

Figure S25: Dynamic formation of emulsions and their penetration into droplets. The aqueous phase is shown in green color, and the surrounding oil phase is black. 3D image of **(a)** DI- water droplet on a solid surface submerged in oil, **(b)** water droplet in 20.% Span micellar solution, and **(c)** 4.0 wt.% silica dispersion droplet in Span micellar solution. **(d)** A slice (1/4) of 3D image of the silica droplet in Span solution **(c)** at three time scales (5, 15, and 30 minutes). Over time, emulsions penetrate into the depth of the droplet.

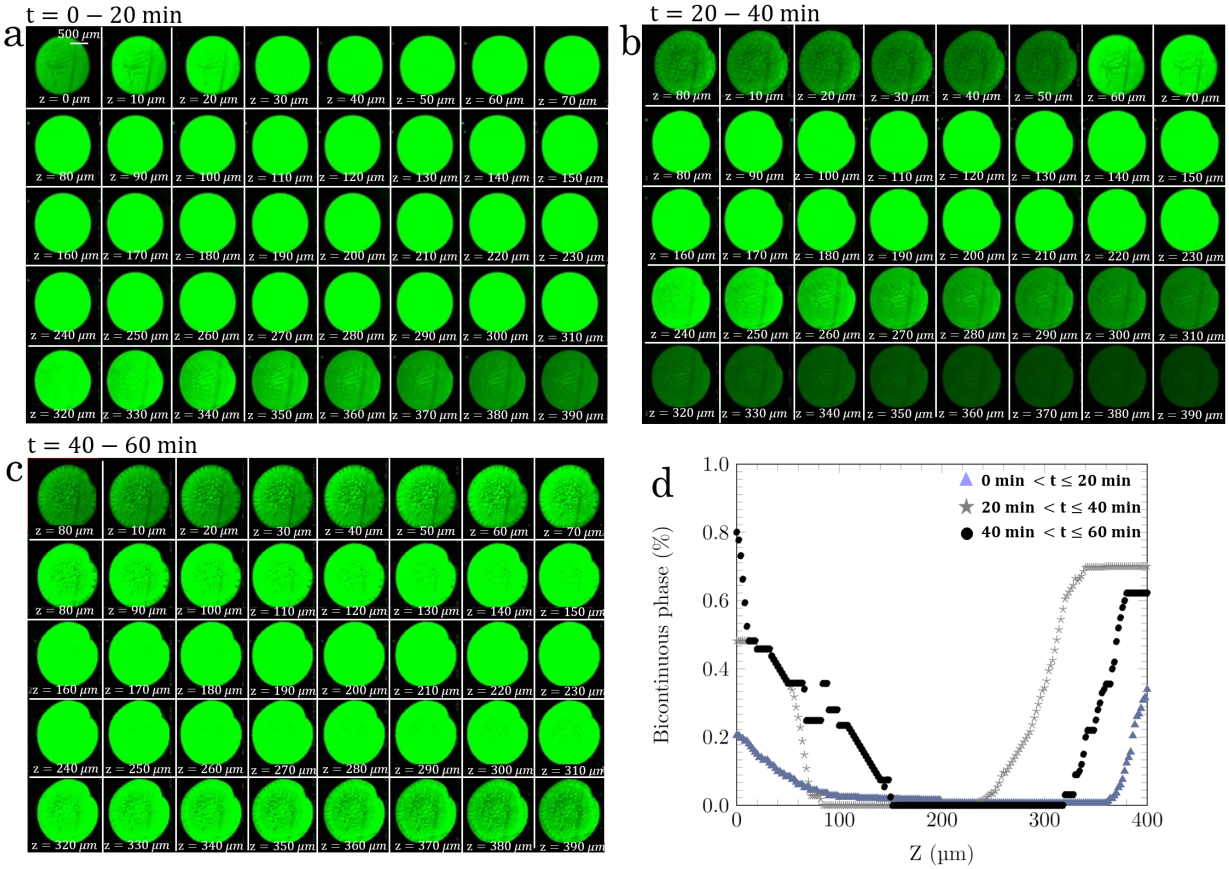

Figure S26: Z-stack images of 3D confocal DI water droplet in 20.0 wt.% Span micellar solution. Capturing images from bottom to the top of the droplet (400 μm) takes 20 minutes. Thus images are shown at 20 minutes time intervals in **(a)**, **(b)**, and **(c)**. Images are shown every 10 μm from bottom to the top of the droplet. **(d)** The coverage of the emulsion phase is calculated from the z-stack images at each time interval. Source data are provided as a Source Data file.

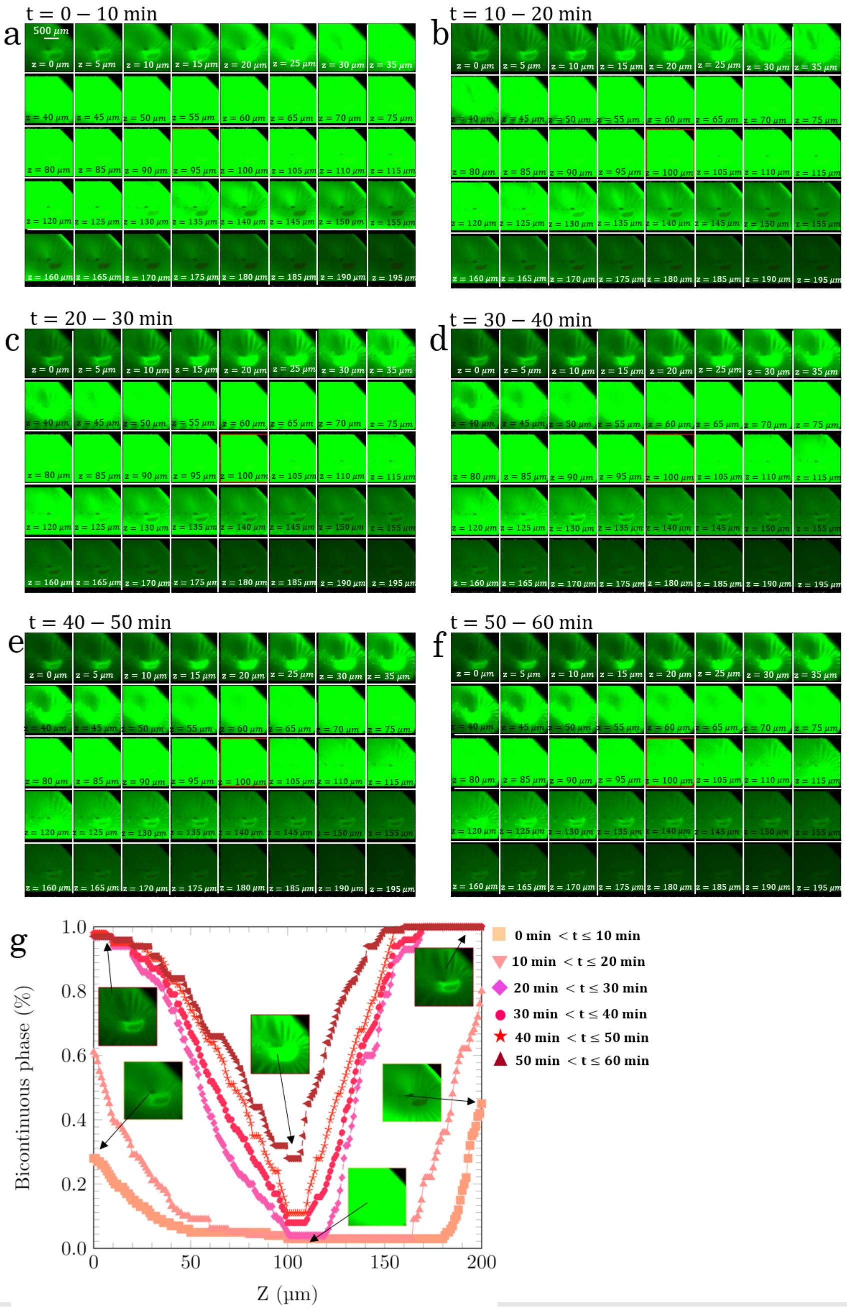

Figure S27: Z-stack images of 3D confocal 4.0 wt.% silica dispersion droplet in 20.0 wt.% Span micellar solution. Capturing images from bottom to the top of the droplet (200 μm) takes 10 minutes. Thus images are shown at 10 minutes time intervals in **(a)**, **(b)**, and **(c)**. Images are shown every 5 μm from bottom to the top of the droplet. **(d)** The coverage of the emulsion phase is calculated from the z-stack images at each time interval. Source data are provided as a Source Data file.

The volume of the emulsified zone divided by the volume of the droplet, i.e., porosity, increases sharply in the first 25 minutes and then it increases slowly over the next 35 minutes (**Fig. S28**, left axis in black). **Fig. S28** shows that the porosity rapidly increases up to 25 minutes while in **Fig. 2c** the intensity of emulsions reaches a maximum within 4 minutes. The inconsistency in the rate of emulsion formation in these figures is due to the differences in the nature of measurements. The rates in **Fig. 2c** are obtained only by analyzing the data on one single layer (from the 2D image) while the porosity (in **Fig. S28**) is calculated by 3D re-constructing the droplet volume. Furthermore, we quantify the 3D images in **Fig. S25d** and evaluate the depth (L_D) at which the emulsification has propagated from the top layer of drop towards the drop center which is growing according to $L_D \sim t^{0.52}$ (**Fig. S28**, right axis in blue).

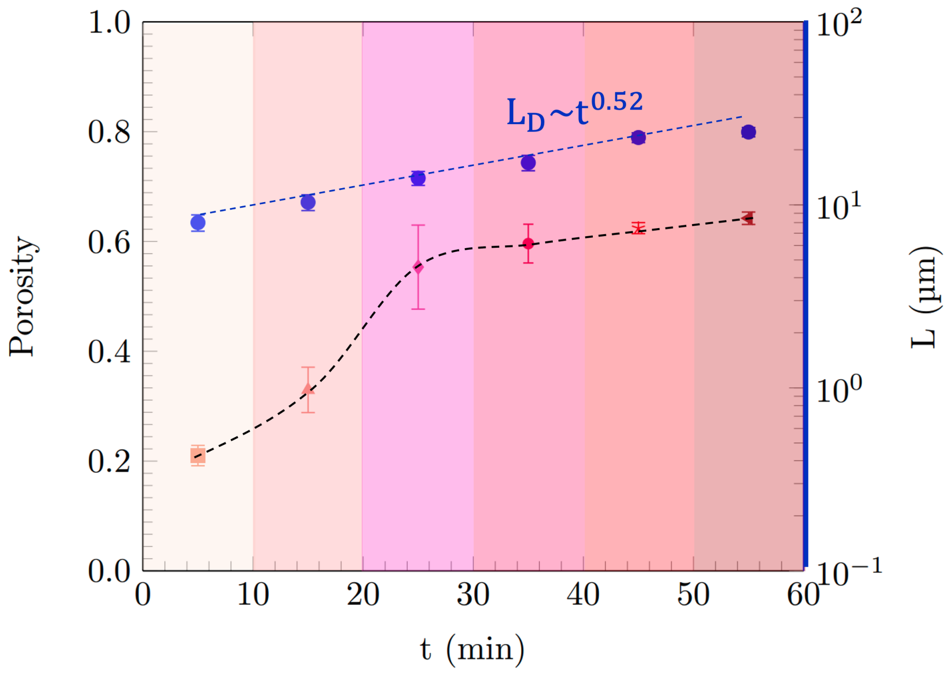

Figure S28: Penetration depth of emulsion over time, left axis in blue. Porosity of 4.0 wt.% silica dispersion droplet in 20.0 wt.% Span micellar solution over time, right axis. The error bars represent the standard deviation of three measurements. Source data are provided as a Source Data file.

Supplementary Note 10: Spongy structured liquids in comparison with other liquid-in-liquid printing techniques

The use of colloidal particles to shape liquid-fluid interfaces in non-equilibrium configurations has attracted significant attention since early 2000 [10–12], and it has been recently extended for liquid-in-liquid printing systems [13–15]. The developed techniques for liquid-in-liquid printing include (i) nanoparticle-polymer jamming at the interface [13–15] and (ii) the formation of lamella structures [16]. Although significant progress has been made so far, current approaches

for all-in-liquid printing have shortcomings that hinder their utilization in real-world applications. These approaches are based on generating a viscoelastic interfacial layer, however, one of the key limitations is the lack of internal structures that could be used for establishing the sophisticated collective communications in biological systems, energy harnessing and storage, design of microreactors, and transport of incompatible species in drug delivery systems. Alternatively, emulsion inks are ideal candidates for creating porous textures and enabling incompatible cargo encapsulation in air [17, 18]. Yet their utilization in a liquid medium is a challenge and it is limited to the complicated drop-by-drop placement techniques [19] or the use of sticky emulsions [20]. The present report bridges between 3D printed solid materials with emulsions and liquid printing systems.

The alternative approach to form structured liquid-in-liquids is using surfactant assemblies in combination with fatty acids and the change of micelle structure [16, 21, 22]. Upon the interaction of spherical micelles of cationic surfactant with fatty acids, the micelle morphology is changed from spherical to lamella and a gel phase is formed at the interface. Niroobakhsh et al. studied the influence of in-situ generated gel on the Rayleigh-Plateau instability and dripping to jetting transition [16] and recently showed its application in 3D printing [22]. Different states of two-phase flow from droplets to straight liquid columns are observed depending on the injection flow rates, surfactant concentrations, and viscosities of the aqueous and oil phases. The liquid filament is formed at the highest surfactant concentration and an intermediate range of flow rates [16, 21]. If the aqueous surfactant solution contains photocurable polymers, the internal phase contains internal nanostructures after the photopolymerization is applied [22].

In this work, we print an aqueous phase into an oil media and spontaneously emulsify the

printed texture. This approach opens up new avenues for further development in all-in-liquid materials. In the following, we summarize the advantages and shortcoming of the current liquid-in-liquid printing approaches and compare them with our developed method.

Table S2: Summary of current liquid-in-liquid 3D printing methods and their comparison with spongy liquid columns.

Method	Publications	Materials	Advantages	Shortcomings
Nanoparticle-polymer jamming	Toor2017	COOH-Silica/PDMS-NH2	1. Spontaneous formation of a viscoelastic interfacial layer.<br>2. Fluid exchange between the printed frame and surrounding media<br>3. Can be used in many particle-polymer systems<br>4. Creating multi-layer all-in-liquid fluid channel that enhances the biocompatibility	1. Incapability of encapsulating incompatible cargoes (particles) inside the printing frame<br>2. Lack of internal structure<br>3. Long-time stability has not been reported
	Lui2017	CNC-OSO3/PS-NH2		
	Shi2018	CNC-OSO3/PS-NH2		
	Forth2018	COOH-Silica, COOH-Au, COOH-CNC /NH2-PDMS-NH2, PDMS-NH2, copolymer		
	Feng2019	Nanoclay/ NH2-PDMS-NH2		
	Toor2019	COOH-Silica/ NH2-PDMS-NH2, PDMS-NH2		
	Qian2020	DNA/ POSS-NH2		
	Sun2020	α-CD-Au/ Azo-PS, Azo-PLLA		
	Wang2022	SPAA/ Ad-PLLA		
	Yin2021	CNC/PDADMAC		
	Kamkar2022	GO/ POSS-NH2		
	Lui2022	CNC/POSS-NH2		
Formation of lamella structures	Niroobakhsh2018	CPCI surfactant/oleic acid	Spontaneous formation of a viscoelastic interfacial layer	1. Incapability of encapsulating incompatible cargoes (particles) inside the printing frame<br>2. Lack of internal structure<br>3. Long-time stability can only be achieved by photopolymerization<br>4. Requires change in micelle morphology
	Niroobakhsh2019	CPCI surfactant/oleic acid		
	Honaryar2021	CPCI- PEGDA, F68-PEGDA/oleic acid		
Emulsion printing in liquid	Zhao2021	Adhesive oil in water emulsions are stabilized with copolymer surfactants functionalized by zwitterionic and ternary amine groups.	1. Formation of porous internal structures<br>2. Encapsulation of incompatible materials	1. Requires input energy for formation of emulsions<br>2. It is limited to the gel like emulsions<br>3. Conventional emulsions suffer from the stability
	Kamkar 2022	Oil in water emulsions are stabilized with GO and are printed an oil phase that contains POSS		
Spongy liquid columns			1. Formation of porous internal structures<br>2. Does not require input energy for emulsification<br>3. Interactions between the printed texture and surrounding media<br>4. Spontaneous formation of a viscoelastic interfacial layer<br>5. Encapsulation of incompatible materials<br>1. Printed structure remains stable for up to two months	2. Requires the presence of surfactant micelles<br>3. Requires nanoparticles to reduce the interfacial tension to values lower than 0.1 mN/m

References

[1] R. Darby, R. Darby, and R. P. Chhabra, *Chemical engineering fluid mechanics, revised and expanded*. CRC Press, 2017.

[2] M. Schmitt, R. Toor, R. Denoyel, and M. Antoni, "Spontaneous microstructure formation at water/paraffin oil interfaces," *Langmuir*, vol. 33, no. 49, pp. 14011–14019, 2017.

[3] P. S. Silva, S. Zhdanov, V. M. Starov, and R. G. Holdich, "Spontaneous emulsification of water in oil at appreciable interfacial tensions," *Colloids and Surfaces A: Physicochemical and Engineering Aspects*, vol. 521, pp. 141–146, 2017.

[4] K. A. Macmillan, J. R. Royer, A. Morozov, Y. M. Joshi, M. Cloitre, and P. S. Clegg, "Rheological behavior and in situ confocal imaging of bijels made by mixing," *Langmuir*, vol. 35, no. 33, pp. 10927–10936, 2019.

[5] J. W. Tavacoli, J. H. Thijssen, A. B. Schofield, and P. S. Clegg, "Novel, robust, and versatile bijels of nitromethane, ethanediol, and colloidal silica: Capsules, sub-ten-micrometer domains, and mechanical properties," *Advanced Functional Materials*, vol. 21, no. 11, pp. 2020–2027, 2011.

[6] P. Bazazi and S. H. Hejazi, "Spontaneous formation of double emulsions at particle-laden interfaces," *Journal of Colloid and Interface Science*, vol. 587, pp. 510–521, 2021.

[7] P. Guillot, A. Colin, A. S. Utada, and A. Ajdari, "Stability of a jet in confined pressure-driven biphasic flows at low reynolds numbers," *Physical Review Letters*, vol. 99, no. 10, p. 104502, 2007.

[8] S. D. Geschiere, I. Ziemecka, V. van Steijn, G. J. Koper, J. H. v. Esch, and M. T. Kreutzer, "Slow growth of the rayleigh-plateau instability in aqueous two phase systems," *Biomicrofluidics*, vol. 6, no. 2, p. 022007, 2012.

[9] S. Tomotika, "On the instability of a cylindrical thread of a viscous liquid surrounded by another viscous fluid," *Proceedings of the Royal Society of London. Series A-Mathematical and Physical Sciences*, vol. 150, no. 870, pp. 322–337, 1935.

[10] K. Stratford, R. Adhikari, I. Pagonabarraga, J.-C. Desplat, and M. E. Cates, "Colloidal jamming at interfaces: A route to fluid-bicontinuous gels," *Science*, vol. 309, no. 5744, pp. 2198–2201, 2005.

[11] E. M. Herzig, K. White, A. B. Schofield, W. C. Poon, and P. S. Clegg, "Bicontinuous emulsions stabilized solely by colloidal particles," *Nature Materials*, vol. 6, no. 12, pp. 966–971, 2007.

[12] A. B. Subramaniam, M. Abkarian, and H. A. Stone, "Controlled assembly of jammed colloidal shells on fluid droplets," *Nature Materials*, vol. 4, no. 7, pp. 553–556, 2005.

[13] M. Cui, T. Emrick, and T. P. Russell, "Stabilizing liquid drops in nonequilibrium shapes by the interfacial jamming of nanoparticles," *Science*, vol. 342, no. 6157, pp. 460–463, 2013.

[14] A. Toor, B. A. Helms, and T. P. Russell, "Effect of nanoparticle surfactants on the breakup of free-falling water jets during continuous processing of reconfigurable structured liquid droplets," *Nano Letters*, vol. 17, no. 5, pp. 3119–3125, 2017.

[15] W. Feng, Y. Chai, J. Forth, P. D. Ashby, T. P. Russell, and B. A. Helms, "Harnessing liquid-in-liquid printing and micropatterned substrates to fabricate 3-dimensional all-liquid fluidic devices," *Nature Communications*, vol. 10, no. 1, pp. 1–9, 2019.

[16] Z. Niroobakhsh, J. A. LaNasa, A. Belmonte, and R. J. Hickey, “Rapid stabilization of immiscible fluids using nanostructured interfaces via surfactant association,” *Physical Review Letters*, vol. 122, no. 17, p. 178003, 2019.

[17] M. R. Sommer, L. Alison, C. Minas, E. Tervoort, P. A. Rühs, and A. R. Studart, “3d printing of concentrated emulsions into multiphase biocompatible soft materials,” *Soft Matter*, vol. 13, no. 9, pp. 1794–1803, 2017.

[18] S. Roh, D. P. Parekh, B. Bharti, S. D. Stoyanov, and O. D. Velev, “3d printing by multiphase silicone/water capillary inks,” *Advanced Materials*, vol. 29, no. 30, p. 1701554, 2017.

[19] G. Villar, A. D. Graham, and H. Bayley, “A tissue-like printed material,” *Science*, vol. 340, no. 6128, pp. 48–52, 2013.

[20] J. Zhao, Z. Pan, D. Snyder, H. A. Stone, and T. Emrick, “Chemically triggered coalescence and reactivity of droplet fibers,” *Journal of the American Chemical Society*, vol. 143, no. 14, pp. 5558–5564, 2021.

[21] Z. Niroobakhsh and A. Belmonte, “Dynamics of a reactive micellar oil-water interface in a flowing liquid column,” *Journal of Non-Newtonian Fluid Mechanics*, vol. 261, pp. 111–122, 2018.

[22] H. Honaryar, J. A. LaNasa, E. C. Lloyd, R. J. Hickey, and Z. Niroobakhsh, “Fabricating robust constructs with internal phase nanostructures via liquid-in-liquid 3d printing,” *Macromolecular Rapid Communications*, vol. 42, no. 22, p. 2100445, 2021.
