# Supplementary material for: Spongy all-in-liquid materials by in-situ formation of emulsions at oil-water interfaces
Source: Nat Commun. 2022 Jul 18;13:4162. doi: 10.1038/s41467-022-31644-2 (PMC9293904; doi:10.1038/s41467-022-31644-2)
Supplement: Supplementary file 3 — Description of Additional Supplementary Files [file 41467_2022_31644_MOESM3_ESM.pdf]

Description of Additional Supplementary Files

File Name: Supplementary Movie 1
Description: In this regime, single drops detach from the injection needle tip and sediment through the micellar solution

File Name: Supplementary Movie 2
Description: The BOAS regime appears where the detached drops merge with the preceding drops, and is identified with the dominant wavelength of 1.9 mm < λ_{mean} < 2.6 mm.

File Name: Supplementary Movie 3
Description: In the connected regime, a thread connects the neighbouring droplets, i.e., a combination of column and BOAS shapes, with wavelengths of 2.5 mm < $\lambda_{cut-off}$ < 3.2 mm

File Name: Supplementary Movie 4
Description: In the column state, which occurs for intermediate injection speeds, the injected liquid forms a stable thread-like shape without breaking into droplets, where the cut-off wavelength is 0.7 mm < $\lambda_{cut-off}$ < 1.0 mm.

File Name: Supplementary Movie 5
Description: It shows the expansion of interfacial area by the penetration of emulsions from the interface to the oil phase.

File Name: Supplementary Movie 6
Description: The stability and durability of the liquid filaments after the injection has ceased. The 4.0 wt.% silica dispersion is injected in a 20.0 wt.% micellar solution forming a stable column. Once the injection is stopped, the liquid inside the column drains immediately leaving a wrinkled tube skin. The generated emulsion phase forms a viscoelastic skin keeping the structure of the column intact. Upon re-injecting the liquid, the column is restored to its original shape.

File Name: Supplementary Movie 7
Description: A tortuous channel of silica dispersion is printed manually in a Span micellar solution. Then, the printed channel is punched by the tip of a second micropipette and 10 µl of dyed DI water (yellow) is injected inside the channel.
